# The role of nutritional supplementation in preventing postoperative complications: a systematic review and meta-analysis

**DOI:** 10.3389/fnut.2026.1744249

**Published:** 2026-02-19

**Authors:** Marina Sartini, Filippo Del Puente, Alessio Carbone, Martino Oliva, Sara Pessano, Marcello Feasi, Carolina Piccinini, Elisa Schinca, Gianluca Ottria, Maria Luisa Cristina

**Affiliations:** 1 Operating Unit Hospital Hygiene, Galliera Hospital, Genoa, Italy; 2 Department of Health Sciences, University of Genoa, Genoa, Italy; 3 Department of Infectious Diseases, Galliera Hospital, Genoa, Italy; 4 Division of Medical Oncology, Department of Medicine, Galliera Hospital, Genoa, Italy; 5 Scientific Direction, Clinical Epidemiology Unit, IRCCS Giannina Gaslini, Genoa, Italy; 6 Department of Public Health, Experimental and Forensic Medicine, Unit of Biostatistics and Clinical Epidemiology, Medical Statistics and Biometry Postgraduate School, Pavia, Italy

**Keywords:** blood-borne Infections, diet, meta-analysis, postoperative complications, surgical wound infection

## Abstract

**Introduction:**

Hospital-acquired infections represent an increasingly urgent issue, particularly in intensive care and surgical settings, also in light of growing concerns about antibiotic resistance. Concurrently, nutrition—due to its immunomodulatory effects—is gaining attention as a key factor across multiple medical disciplines. The aim of this study was to assess perioperative nutritional interventions—immunonutrition, probiotics, synbiotics, and protein integration—in reducing surgical site infections (SSIs), healthcare-associated infections (HAIs) and hospital length of stay (LOS) in surgical patients.

**Methods:**

A comprehensive search on principal databases was performed on December 2024. As effect measures, was calculated the Odds Ratios (OR). The assessment of potential bias and the evaluation of study quality was conducted. Thirty-nine publications were selected for inclusion in the meta-analysis.

**Results:**

Immunonutrition was associated with a reduction in infectious complications (OR = 0.36) and showed a probable reduction in SSIs (OR = 0.35). Some benefits may also be obtained with the administration of probiotics (OR = 0.61). Bloodstream infections (BSIs) appeared to be substantially reduced with probiotics (OR = 0.42), although the certainty of evidence was low. Analytical studies showed weaker effects than RCTs of immunonutrition on SSI reduction compared with randomized control trials (RCTs), underscoring the importance of RCT evidence for clinical recommendations.

**Discussion:**

Based on RCT evidence, immunonutrition demonstrates robust efficacy in preventing SSIs with moderate certainty evidence, and appears ready for clinical implementation in high-risk surgical setting. Probiotics showed moderate effects on selected infectious outcomes, supported by low-certainty evidence, and may be considered as complementary interventions. Protein supplementation demonstrated statistical significance; however, the very low certainty of evidence precludes firm recommendations. Clinical implementation should prioritize interventions supported by RCT evidence within multimodal prevention strategies. Future research should address heterogeneity in nutritional formulations, standardize outcome definitions, and evaluate real-world effectiveness.

**Systematic review registration:**

https://www.crd.york.ac.uk/prospero/.

## 1 Introduction

HAIs represent a significant global burden causing numerous attributable death and Disability-Adjusted Life Years (DALYs) annually (1). Among these, postoperative infections—including SSIs, nosocomial pneumonia, sepsis, and UTIs—are a major challenge, with SSIs significantly increasing hospitalization duration, costs, and mortality (2, 3). Despite advancements, their persistence underscores the need for adjunctive strategies. In this manuscript, we use “postoperative infections” as an umbrella term encompassing HAIs occurring after surgery, including SSIs, BSIs, urinary tract infections (UTIs), and pneumonia. While these infections may occur in the postoperative period, they represent distinct clinical entities with potentially different surveillance definitions across studies.

Recently, nutrition has emerged as a modifiable factor to modulate immune function and gut microbiota. Interventions such as immunonutrition, probiotics, synbiotics, and protein integration have been investigated to reduce infection rates and hospital LOS. Immunonutrition, which involves supplementation with immune-modulating nutrients such as arginine, omega-3 fatty acids, and nucleotides, has been hypothesized to enhance immune responses and clinical outcomes, particularly in critically ill or surgical patients (4). Probiotics, live microbial supplements, and synbiotics (combinations of probiotics and prebiotics, non-digestible sugars that favor specific bacterial strains’ growth) aim to restore gut microbiota balance, a key determinant of systemic immunity and infection susceptibility (5). Protein supplementation addresses catabolic stress associated with hospitalization and critical illness (6), supporting tissue repair and immune function (4).

However, systematic evidence is inconsistent. While some meta-analyses suggest reduced HAI rates with immunonutrition (7), others emphasize significant heterogeneity in studies (8). Similarly, trials on probiotics and synbiotics yield mixed results, with concerns about strains, dosing, and methodological quality (9). Protein integration remains underexplored. Critical limitations include underpowered studies, publication bias (10), and limited generalizability.

This systematic review and meta-analysis synthesizes current evidence on these nutritional interventions for HAI prevention and LOS reduction. By addressing methodological limitations and heterogeneity, we aim to provide a comprehensive evaluation to inform clinical practice and future research priorities.

## 2 Methods

This systematic review and meta-analysis followed the PRISMA Guideline (11) to ensure methodological soundness and transparency. The research question was structured using the PICO framework. The study protocol was registered in the PROSPERO database (CRD42024575184).

### 2.1 Data sources and search strategy

A systematic literature search was conducted in PubMed/MEDLINE, Scopus, and Cochrane up to December 2024. We also searched Google Scholar to increase search sensitivity, manually screening the first 200 results and carefully excluding duplicates and low-quality sources that did not meet our inclusion criteria. We searched the following keywords and Medical Subject Headings (MeSH): “Preoperative assessment” AND “diet” AND “infection,” “Prevention” AND “healthcare-associated” AND “infections” AND “diet,” (“Prevention of HAIs” AND “diet”), (prevention surgical infection [MeSH Terms]) combined with specific terms (glutamine [MeSH Terms], milk [MeSH Terms], arginine [MeSH Terms], Vitamin [MeSH Terms], Nutrient [MeSH Terms], Selenium [MeSH Terms], zinc [MeSH Terms], Glycine [MeSH Terms], probiotic [MeSH Terms]). MeSH terms were selected following the National Center for Biotechnology Information (NCBI) guidelines, with wild-card operators employed when appropriate. No backward or forward citation tracking (snowballing) was performed.

### 2.2 Selection of studies

Inclusion criteria: (1) exploring the effect of immunonutrition, synbiotics, probiotics and protein integration on HAI prevention in surgical patients; (2) reporting clinically relevant outcomes such as Infectious complication, BSI, SSIs, UTI, Pneumonia, LOS; (3) retrospective cohort studies, prospective cohort studies, single-center or multicenter prospective studies, RCT, or case-control studies.

Exclusion criteria: (1) studies not directly addressing the research question; (2) articles lacking sufficient quantitative data; (3) case reports, editorials, narrative reviews, feasibility studies, pilot studies or commentaries; (4) studies that did not meet the following PICOS criteria (Table 1):

- Population: Patients undergoing surgery.
- Intervention: Perioperative nutritional interventions (probiotics, synbiotics, immunonutrition, protein integration).
- Comparators: Patients receiving standard nutrition, conventional therapy, no treatment, or placebo.
- Outcomes: Infectious complication, SSI, BSI, UTI, Pneumonia, LOS.
- Study Design: RCT, cohort studies, and case–control studies.

**Table 1:** Search strategy adopted in the present systematic review and meta-analysis.

Search strategy	Details	
Search string	(“Preoperative assessment” AND “diet” AND “infection”)-(“prevention” AND “healthcare-associated” AND “infections” AND “diet”)-(“prevention of HAIs” AND “diet”)-(prevention surgical infection [MeSH terms]) AND ((glutamine [MeSH terms] OR milk [MeSH terms] OR arginine [MeSH terms] OR vitamin [MeSH terms] OR Nutrient [MeSH terms] OR selenium [MeSH terms] OR zinc [MeSH terms] OR glycine [MeSH terms] OR probiotic [MeSH terms])).	
Inclusion criteria	P (patients/population)	Surgery patients
	I (intervention/exposure)	Patients who received probiotics, synbiotics, immunonutrition, or protein integration before surgery
	C (comparisons/comparators)	Patients who received regular food, standard therapy, no therapy, or placebo
	O (outcome)	Primary outcomes: infectious complication, SSI, BSI
		Secondary outcomes: UTI, pneumonia, hospital LOS
	S (study design)	Randomized controlled trials (RCTs), cohort studies and case–control studies
Databases	PubMed/MEDLINE, Scopus, Cochrane and Google Scholar	
Exclusion criteria	Items not directly relevant to the research question; studies did not have sufficient information and data available to be analyzed; articles did not meet the PICOS criteria	
Time filter	From inception to December 2024	
Language filter	None (any language)	

No limits were set on publication date or language. The oldest included study was published in 1999, thus our evidence base spans from 1999 to December 2024.

### 2.3 Data extraction and risk of bias assessment

Following the title and abstract screening, potentially relevant articles were retrieved in full and assessed for eligibility criteria. This selection process was carried out independently by four reviewers, conflicts have been solved with the help of a fifth reviewer when needed. A standardized data extraction form was used to collect information on study design, population characteristics, type and dosage of supplements, primary and secondary outcomes, and follow-up duration.

The methodological quality and potential risk of bias of the included studies were assessed independently by four researchers using National Institutes of Health (NIH) Quality Assessment Tool (12) tailored to each study type.

Of the included RCT, 16 publications were classified as having “Low risk of bias,” 9 “Moderate risk of bias” and on “High risk of bias.” Among the analytical studies, 6 publications were identified as having “Low risk of bias,” 4 “Moderate risk of bias,” and 3 “High risk of bias.” Results were displayed graphically using the Traffic Light Plot (Supplementary Figure 1) and a Summary Plot (Supplementary Figure 2), respectively. Blinding bias was not deemed critical, as outcomes were objective.

### 2.4 Grade assessment

GRADE approach (13) was used to assess the certainty of the evidence. Two reviewers independently assessed the certainty of the evidence and resolved disagreements by discussion; all decisions have been justified and reported in the footnotes. Results have been reported following the recommendations from the GRADE Guidance (14) and classified in four levels based on the confidence that the true value of the estimate is on one side of a threshold of interest or within a specific range:

- High certainty;
- Moderate certainty;
- Low certainty;
- Very low certainty.

We acknowledge that definitions of postoperative infections (SSI, BSI, UTI, pneumonia) and surveillance methods may vary across included studies, potentially affecting the comparability of pooled effect estimates. This heterogeneity in outcome ascertainment represents an important limitation of our analysis. We used the GRADEpro software^1^ to create the summary of findings table.

### 2.5 Statistical analysis

Both qualitative and quantitative data synthesis were performed. Four researchers independently conducted the synthesis, resolving any discrepancies through discussion and consensus. Meta-analysis was performed using STATA SE 19 (StataCorp LLC, College Station, Texas, United States). While assessing heterogeneity an *I*^2^ value of 25, 50%, or 75% was classified as low, moderate, or high heterogeneity, respectively. A random-effects model was used when heterogeneity was significant; otherwise, a fixed-effects model was applied. Summary outcome measures included OR with 95% confidence intervals (CIs) for binary outcomes and MD for continuous outcomes. For studies reporting continuous outcomes in terms of medians and interquartile ranges, the corresponding mean and standard deviation were estimated using the methods provided by Luo et al. (15) and Wan et al. (16). Sensitivity analyses were performed to evaluate result stability by sequentially excluding individual studies. Publication bias was not assessed because less than 10 studies contributed to each analysis. All statistical analyses were conducted following established guidelines to ensure robustness and reproducibility.

## 3 Results

### 3.1 General description of studies

**Figure 1:**
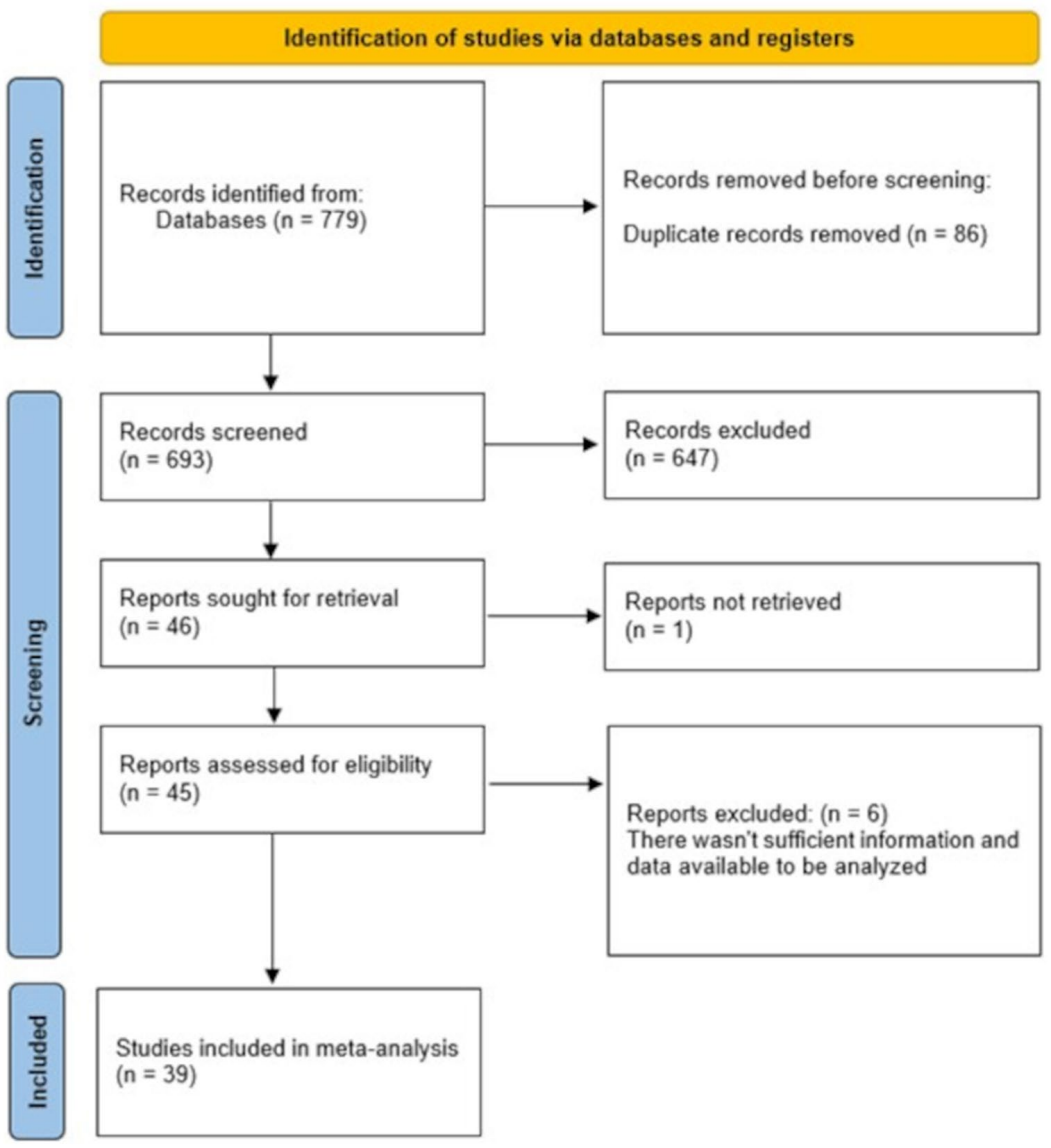
PRISMA 2020 flow diagram of study selection, inclusion, and synthesis.

The meta-analysis included a total of 39 publications [26 RCTs (17–42) and 13 analytical studies (43–55)] (Figure 1) that evaluated the efficacy of nutritional supplements in patients undergoing surgical procedures, primarily oncological interventions. In three of these publications (20, 43, 51), multiple comparisons were reported (each of these studies included two intervention groups and one control group, with the intervention groups differing, for example, in the timing of integration or in the type of surgical procedure) and were therefore analyzed separately in the meta-analysis. Geographically, the studies were distributed as follows: 43.6% in Asia, 33.3% in Europe, 12.8% in North America, and 10.3% in South America.

Regarding surgical approach, 28 studies involved only laparotomic procedures, 5 mixed surgical interventions (including both laparotomic and laparoscopic procedures within the same study), and the remaining 6 referred to other types of surgeries (orthopedic, cardiac, and mixed surgical approaches).

The mean age of patients was relatively evenly distributed, with 53.9% of participants younger than 65 years, 33.3% older than 65 years, and 12.8% of unspecified age. The routes of supplement administration were predominantly oral (66.7%), while 12.8% were enteral, 2.6% parenteral, and the remaining 17.9% involved a combination of these modalities.

The main characteristics of the studies included in the meta-analysis are reported in Supplementary Table 1, summarizing study design, location, type of nutritional integration, and participant demographics (sample size, age, and sex). Supplementary Table 2 outlines outcome data, showing the absolute and relative frequencies of infectious complications, SSIs, BSIs, UTIs, and pneumonia in both intervention and control groups. These tables provide a clear comparison of effect estimates, reflecting variations in study populations, integration protocols, and outcome definitions.

Regarding the maturity of evidence and clinical adoption, immunonutrition represents the most established intervention with relatively standardized formulations and established clinical protocols, particularly in oncological surgery. Probiotics and synbiotics remain more experimental, with substantial heterogeneity in strain selection, dosing regimens, and administration protocols across studies. Protein supplementation is the least studied intervention with limited data available.

Summary effect estimates for each integration type, along with subgroup analyses, are presented in the main text and detailed in Supplementary Tables 3–7.

#### 3.1.1 Infectious complication

##### 3.1.1.1 Randomized controlled trials

Among the 26 RCTs, 13 studies evaluated the effect of nutritional integration on general infectious complications: 5 studies (17, 18, 21, 33, 40) assessed immunonutrition, 2 studies (25, 27) probiotics, 5 studies (23, 26, 28, 32, 34) synbiotics, and one protein integration (38).

###### 3.1.1.1.1 Immunonutrition

**Figure 2:**
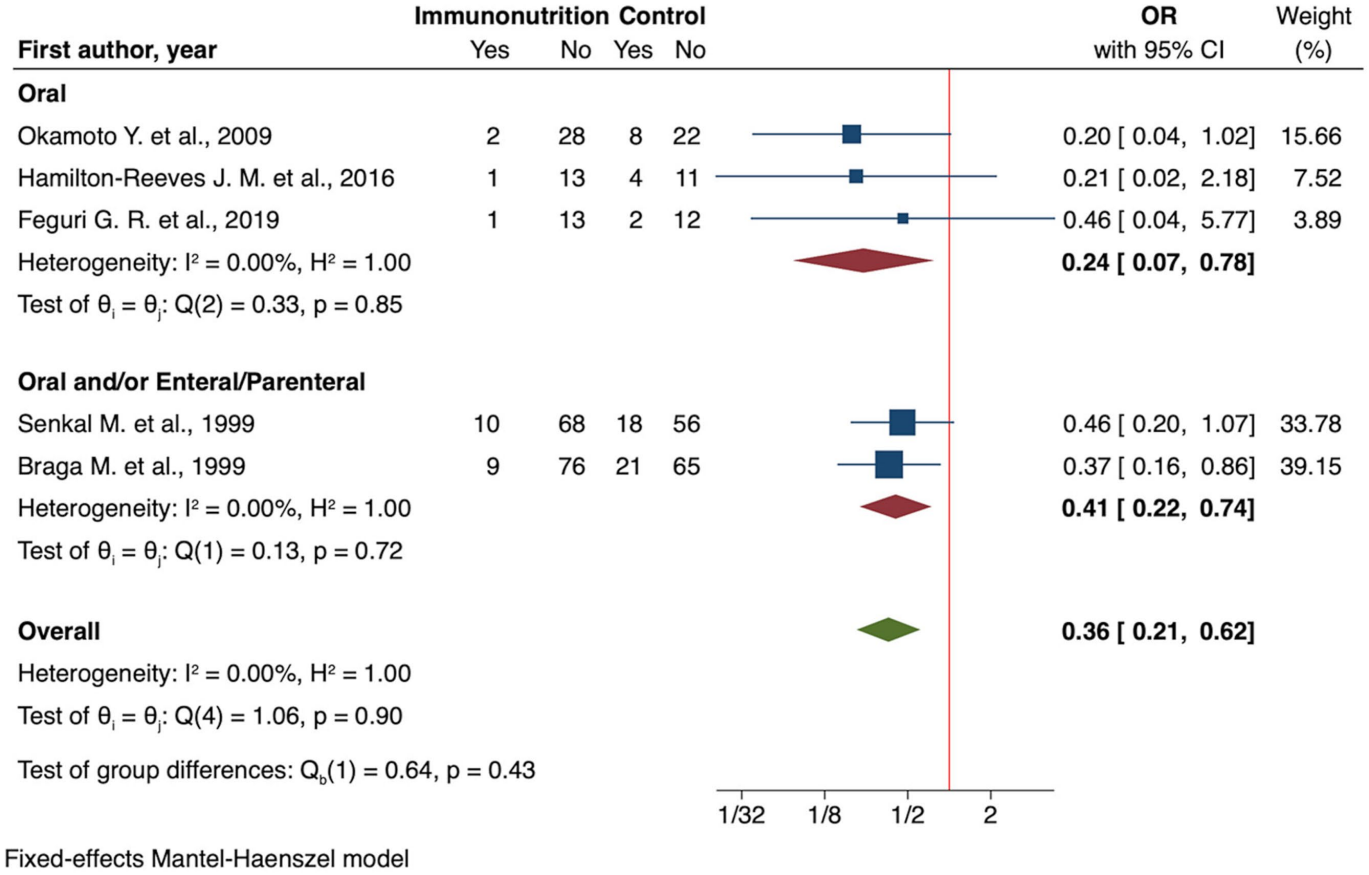
Forest plot showing the effect of immunonutrition vs. no intervention (control) on infectious complications by route of administration for RCT studies.

Meta-analysis of RCTs demonstrated a significant reduction in infectious complications compared to standard nutritional care (OR 0.36, 95% CI: 0.21 to 0.62; *I*^2^ 0.00% Figure 2), low certainty of evidence (Table 2). Subgroup analyses revealed the following findings:

**Table 2:** Nutritional interventions compared to standard of care for preventing postoperative infectious complications only for RCT studies.

Outcomes	Interventions	Anticipated absolute effects* (95% CI)		Relative effect (95% CI)	No of participants (studies)	Certainty of the evidence (GRADE)	Comments
		Risk with standard of care	Risk with probiotics				
Infectious complications	Immunonutrition	242 per 1.000	103 per 1.000 (63 to 165)	OR 0.36 (0.21 to 0.62)	440 (5 RCTs)	⨁⨁◯◯ Low^a^^,^^b^	Immunonutrition may result in a large reduction in infectious complications.
	Probiotics	272 per 1.000	165 per 1.000 (46 to 252)	OR 0.53 (0.13 to 0.90)	255 (2 RCTs)	⨁◯◯◯ Very low^c^^,^^d^	Probiotics may have little to no effect on infectious complications but the evidence is very uncertain.
	Synbiotics	326 per 1.000	166 per 1.000 (68 to 350)	OR 0.41 (0.15 to 1.11)	542 (5 RCTs)	⨁◯◯◯ Very low^a^^,^^e^^,^^f^	The evidence is very uncertain about the effect of synbiotics on Infectious Complications
SSI	Immunonutrition	122 per 1.000	46 per 1.000 (23 to 90)	OR 0.35 (0.17 to 0.71)	475 (3 RCTs)	⨁⨁⨁◯ Moderate^b^	Immunonutrition probably results in a large reduction in SSI.
	Probiotics	189 per 1.000	125 per 1.000 (89 to 175)	OR 0.61 (0.42 to 0.91)	830 (7 RCTs)	⨁⨁◯◯ Low^a^^,^^i^	Probiotics may reduce SSI.
	Synbiotics	140 per 1.000	81 per 1.000 (41 to 153)	OR 0.54 (0.26 to 1.11)	614 (5 RCTs)	⨁⨁◯◯ Low^f^^,^^g^	Synbiotics may result in little to no difference in SSI.
	Protein Supplementation	214 per 1.000	89 per 1.000 (44 to 169)	OR 0.36 (0.17 to 0.75)	262 (4 RCTs)	⨁◯◯◯ Very low^a^^,^^f^^,^^h^	Protein Supplementation may reduce SSI but the evidence is very uncertain.
BSI	Probiotics	151 per 1.000	70 per 1.000 (31 to 149)	OR 0.42 (0.18 to 0.98)	518 (5 RCTs)	⨁⨁◯◯ Low^i^^,^^l^	Probiotics may result in a large reduction in BSI.
	Synbiotics	185 per 1.000	189 per 1.000 (118 to 290)	OR 1.03 (0.59 to 1.80)	347 (5 RCTs)	⨁⨁⨁◯ Moderate^f^	Synbiotics likely results in little to no difference in BSI.

*The risk in the intervention group (and its 95% CI) is based on the assumed risk in the comparison group and the relative effect of the intervention (and its 95% CI). CI, Confidence interval; MD, Mean difference; OR, Odds ratio.

a Downgraded by one level for inconsistency because the studies involved different kind of surgeries.

b Downgraded by one level for imprecision for not optimal information size of the studies.

c Downgraded by one level for high heterogeneity and only partially overlapping CIs.

d Downgraded by two levels very serious imprecision for large CIs and population very far from optimal information size.

e Downgraded by two levels for very serious inconsistency. Evidence of moderate heterogeneity (*I*^2^ 71.19%), with poor overlapping of CIs and Yokoyama et al. (28) showing a different effect of the intervention.

f Downgraded by one level for serious imprecision for not optimal information size and CIs including both “important” and “no effect”.

g Downgraded by one level for serious imprecision for some CIs including both “important” and “no effect”.

h Downgraded by two levels for very serious inconsistency with only partial overlapping of CIs and two studies [Yokoyama et al. (28) and Yokoyama et al. (37)] showing a different effect of the intervention.

i Downgraded by one level for high risk of bias in different studies in multiple critical domains.

l Downgraded by one level for inconsistency: high heterogeneity and one of the biggest study, Liu et al. (30), showed a different effect of the intervention.

- Oral administration showed an OR of 0.24 (95% CI: 0.07 to 0.78, Figure 2), while the combined use of oral and/or enteral/parenteral routes resulted in an OR of 0.41 (95% CI: 0.22 to 0.74, Figure 2).
- Timing also played a role, with preoperative integration showing OR of 0.25 (95% CI: 0.06 to 0.98) and preoperative combined with intra/postoperative integration yielding an OR of 0.39 (95% CI: 0.22 to 0.70).

###### 3.1.1.1.2 Probiotics

The meta-analysis showed a non-significant trend toward reducing infectious complications (OR 0.53, 95% CI: 0.13 to 2.09), very low certainty of evidence (Table 2).

###### 3.1.1.1.3 Synbiotics

The meta-analysis displayed a non-significant trend toward a reduction in infectious complications (OR 0.41, 95% CI: 0.15 to 1.11), very low certainty of evidence (Table 2). Notably, subgroup analyses indicated that oral administration was associated with a significant protective effect (OR 0.33, 95% CI: 0.12 to 0.96). Additionally, patients aged ≤65 years demonstrated a significant reduction in infection risk (OR 0.18, 95% CI: 0.08 to 0.42).

###### 3.1.1.1.4 Protein integration

No meta-analysis could be conducted for protein integration due to the availability of only one study.

###### 3.1.1.1.5 Analytical studies

**Figure 3:**
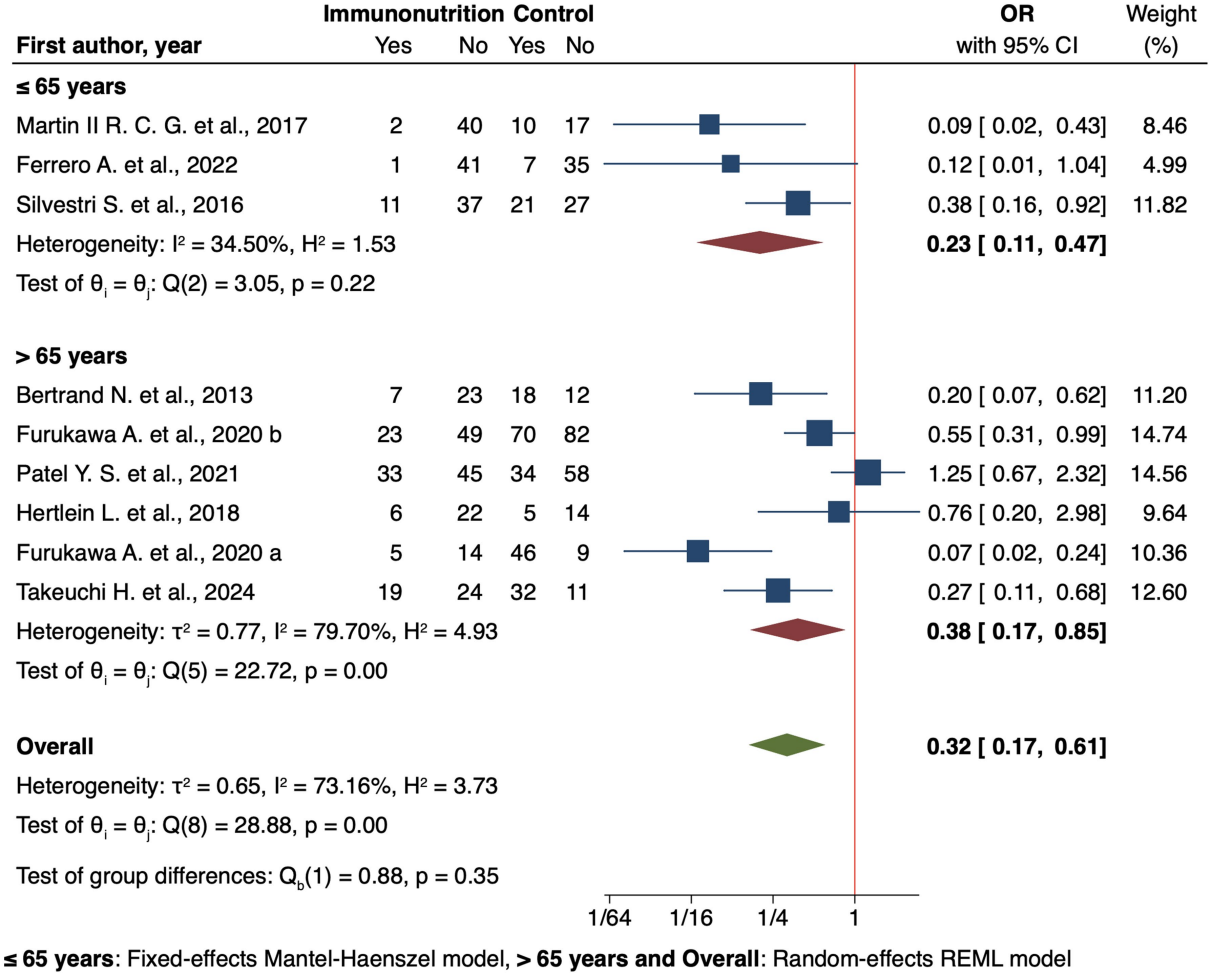
Forest plot showing the effect of immunonutrition vs. no intervention (control) on infectious complications by age for analytical studies.

Eight analytical studies (45–48, 51–53, 55) explored the effect of immunonutrition on infectious complications, one of these (51) contributed with two different populations. Meta-analysis showed a significant protective effect (OR 0.32, 95% CI: 0.17 to 0.61, Figure 3). Subgroup analyses provide additional insights, such as timing, with preoperative supplementation showing an OR of 0.29 (95% CI: 0.14 to 0.58).

Regional differences were also evident, in Europe immunonutrition showed significant protective effects (OR 0.32, 95% CI: 0.18 to 0.58), as well as in Asia (OR 0.24, 95% CI: 0.08 to 0.76). Age-based analyses highlighted a stronger effect on younger patients (≤65 years OR 0.20, 95% CI: 0.07 to 0.59 Figure 3) compared to older patients (>65 years OR 0.38, 95% CI: 0.17 to 0.85 Figure 3).

Lastly, immunonutrition products containing L-arginine, omega-3 fatty acids, RNA nucleotides and soluble fiber (partially hydrolyzed guar gum (PHGG)) had a protective effect (OR 0.26, 95% CI: 0.15 to 0.47).

However, we emphasize that clinical practice recommendations should be based primarily on RCT evidence, given the higher risk of bias and confounding in analytical studies.

#### 3.1.2 Surgical site infection (SSI)

##### 3.1.2.1 Randomized controlled trials

Eighteen studies examined the distribution of nutritional intervention on SSIs: 3 studies (17, 21, 35) examined immunonutrition, 7 studies (23, 25, 27, 29, 30, 36, 41) probiotics, 5 studies (28, 31, 34, 37, 39) synbiotics, and 3 studies (20, 38, 42) protein integration, one of these contributed with two different populations.

###### 3.1.2.1.1 Immunonutrition

**Figure 4:**
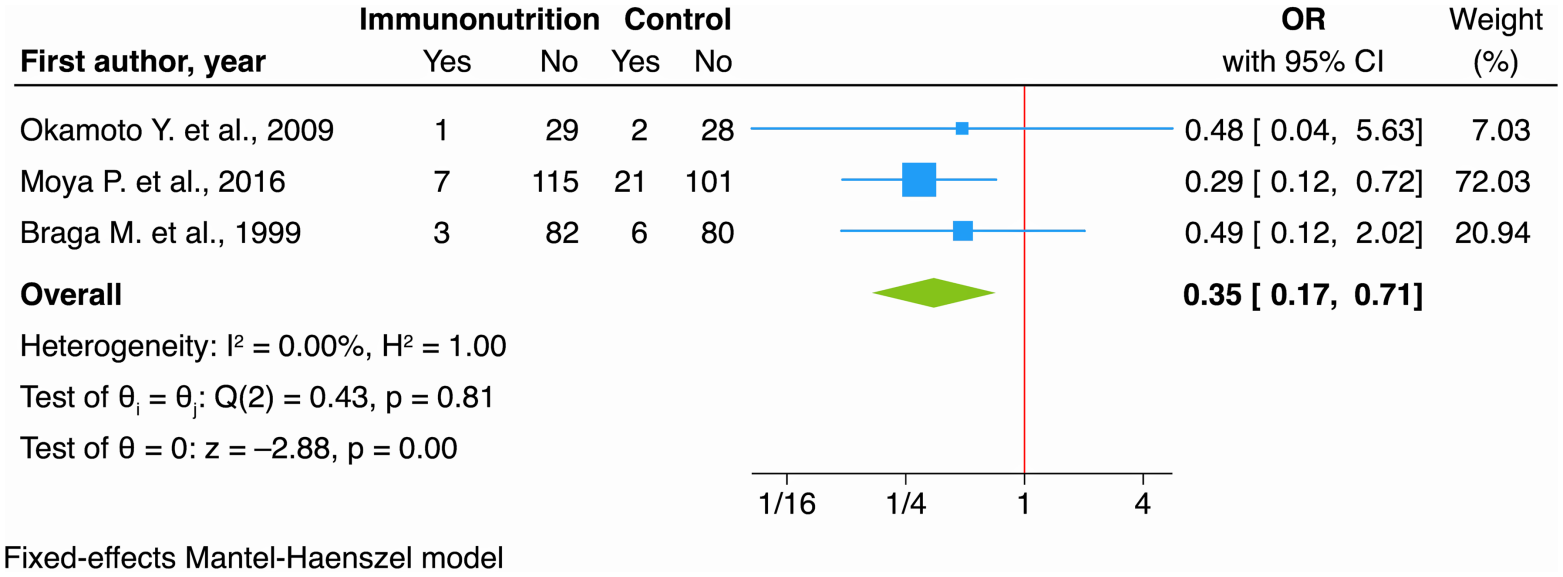
Forest plot showing the effect of immunonutrition vs. no intervention (control) on SSIs for RCT studies.

The meta-analysis indicated a significant reduction with immunonutrition compared to standard nutritional support (OR 0.35, 95% CI: 0.17 to 0.71; *I*^2^ 0.00% Figure 4), moderate certainty of evidence (Table 2).

###### 3.1.2.1.2 Probiotics

**Figure 5:**
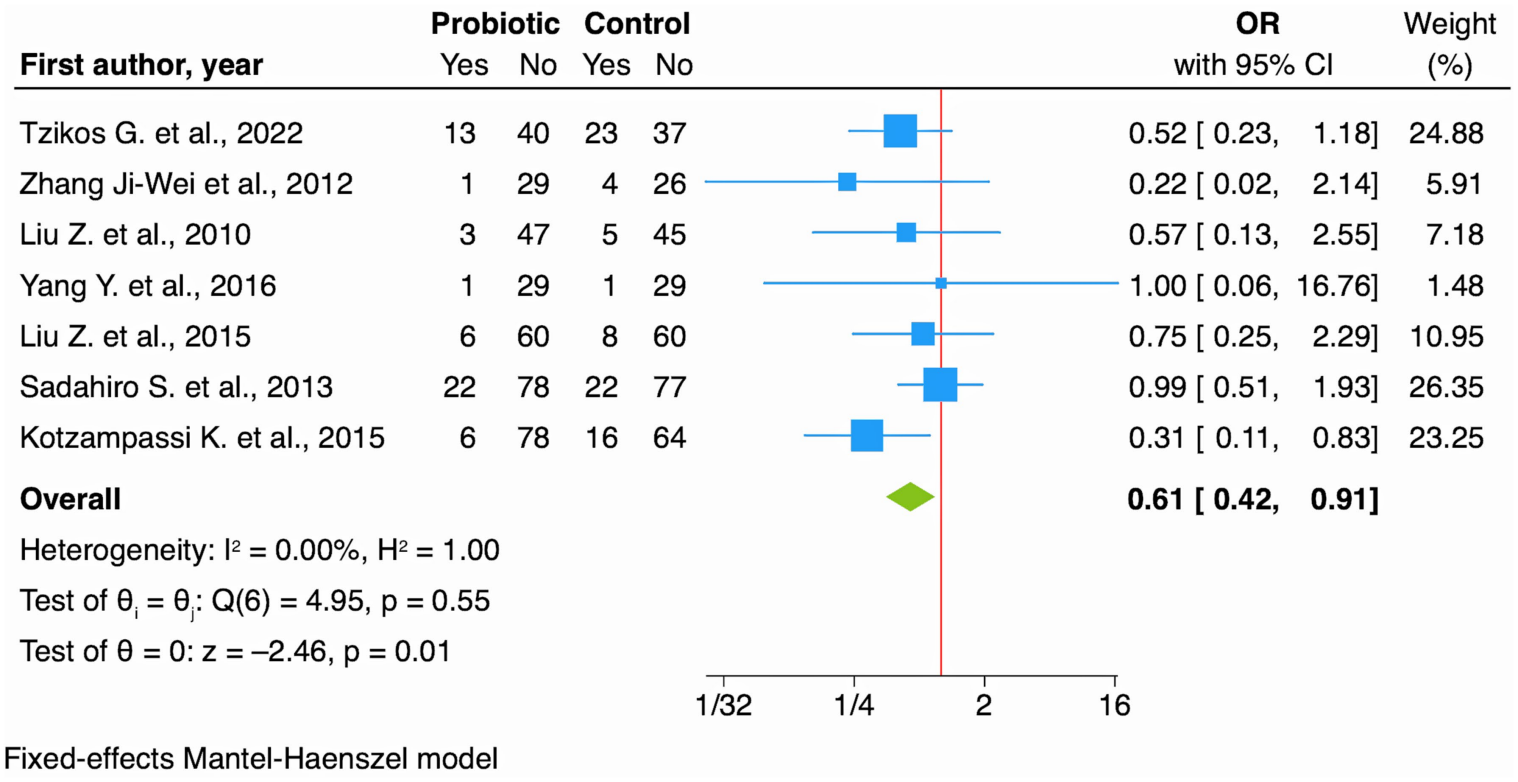
Forest plot showing the effect of probiotics vs. no intervention (control) on SSIs for RCT studies.

The effect of probiotics was protective on SSIs (OR 0.61, 95% CI: 0.42 to 0.91; *I*^2^ 0.00% Figure 5), low certainty of evidence (Table 2). Subgroup analysis by age showed slightly different effects: older patients (>65 years) showed OR of 0.61 (95% CI: 0.32 to 1.15), in the younger group (≤65 years) the OR was 0.66 (95% CI: 0.40 to 1.09). These findings, however, did not reach statistical significance, probably due to the reduced precision of the estimates within the individual subgroups.

###### 3.1.2.1.3 Synbiotics

The meta-analysis suggests a non-significant reduction in SSI risk by 46% (OR 0.54, 95% CI: 0.26 to 1.11), low certainty of evidence (Table 2). This finding is further affected by a non-negligible level of heterogeneity among studies (*I*^2^ 20.26%).

###### 3.1.2.1.4 Protein integration

**Figure 6:**
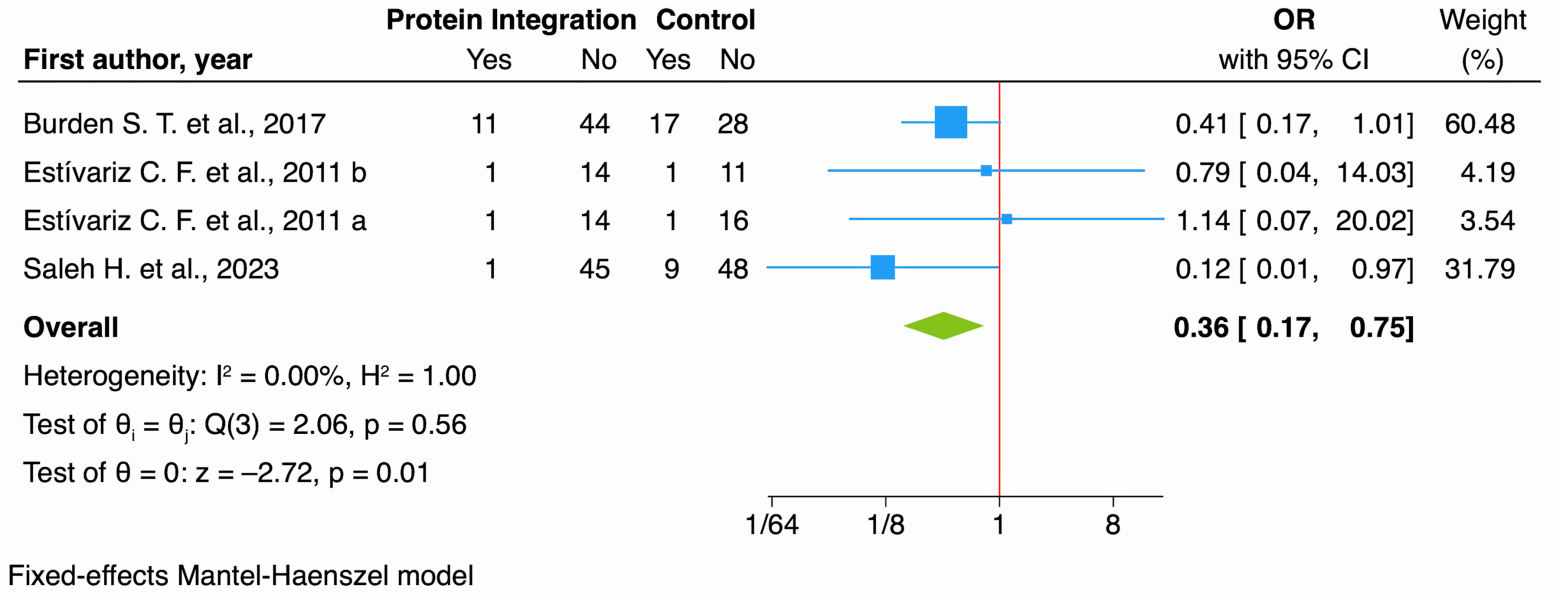
Forest plot showing the effect of protein integration vs. no intervention (control) on SSIs for RCT studies.

The meta-analysis showed a significant risk reduction of 64% (OR 0.36, 95% CI: 0.17 to 0.75; *I*^2^ 0.00% Figure 6), very low certainty of evidence (Table 2). This extremely low certainty substantially limits confidence in the effect estimate and precludes definitive clinical recommendations.

##### 3.1.2.2 Analytical studies

The analysis included 7 analytical studies (43, 46–48, 50, 54, 55), all of which investigated immunonutrition interventions, one of these43 contributed with two different populations.

The meta-analysis of these studies does not confirm the findings from RCTs as it did not show a statistical significance, although it suggests a risk reduction of 42% (OR 0.58, 95% CI: 0.31 to 1.07). Unlike the data from RCTs, the heterogeneity among studies is substantially higher (*I*^2^ 31.37%). Subgroup analysis highlighted that the combination of L-arginine, omega-3 fatty acids, RNA nucleotides and soluble fiber (PHGG) had a better protective effect (OR 0.37, 95% CI: 0.16 to 0.86).

#### 3.1.3 Bloodstream infection (BSI)

##### 3.1.3.1 Randomized controlled trials

Twelve studies examined the effect of nutritional intervention on BSIs: 1 study (17) explored immunonutrition, 5 studies (24, 25, 29, 30, 36) probiotics, 5 studies (19, 26, 28, 31, 37) synbiotics, and one study with two different analysis (20) focused on protein integration.

###### 3.1.3.1.1 Probiotics

The meta-analysis was conducted on probiotics and showed a borderline protective effect (OR 0.42, 95% CI: 0.18 to 0.98, Supplementary Figure 3) with a low to moderate heterogeneity among the studies (*I*^2^ 42.65%) and low certainty of evidence (Table 2).

###### 3.1.3.1.2 Synbiotics

Regarding synbiotics efficacy, the meta-analysis found non-significant effect (OR 1.03, 95% CI: 0.59 to 1.80).

##### 3.1.3.2 Analytical studies

The analysis included 5 analytical studies (43, 44, 46, 48, 49), all of which investigated immunonutrition interventions, one of these43 contributed with two different populations.

The meta-analysis found immunonutrition as protective against sepsis (OR 0.39, 95% CI: 0.25 to 0.61; *I*^2^ 0.00%, Supplementary Figure 4). Subgroup analysis by disease showed a more pronounced effect on non-oncological patients (OR 0.34, 95% CI: 0.16 to 0.73) than in oncological patients (OR 0.46, 95% CI: 0.11 to 1.96).

#### 3.1.4 Urinary tract infection (UTI)

##### 3.1.4.1 Randomized controlled trials

Eight studies examined the distribution of nutritional interventions on UTIs: 1 study (17) examined immunonutrition, 4 studies (24, 29, 30, 36) investigated probiotics, 1 study (31) evaluated synbiotics, and 2 studies (20, 38) focused on protein integration, one of these (20) contributed with two different populations.

###### 3.1.4.1.1 Probiotics

The meta-analysis demonstrated their protective value against UTIs (OR 0.32, 95% CI: 0.14 to 0.74, Supplementary Figure 5) with a low degree of heterogeneity among the studies (*I*^2^ 21.16%) and low certainty of evidence (Supplementary Table 8). Every study that evaluated this particular outcome was designed to administer the drug in the pre-operative period.

###### 3.1.4.1.2 Protein integration

The meta-analysis did not show a protective effect (OR 0.52, 95% CI: 0.22 to 1.27; *I*^2^ 0.00%), very low certainty of evidence (Supplementary Table 8).

#### 3.1.5 Pneumonia

##### 3.1.5.1 Randomized controlled trials

Thirteen studies examined the distribution of nutritional intervention on pulmonary infection: 1 study (17) examined immunonutrition, 5 studies (24, 25, 29, 30, 36) probiotics, 5 studies (26, 28, 31, 37, 39) synbiotics, and 2 studies (20, 38) protein integration. One of these (20) contributed with two different populations.

###### 3.1.5.1.1 Probiotics

The probiotics significantly reduced pulmonary infections (OR 0.42, 95% CI: 0.22 to 0.81; *I*^2^ 0.00%, Supplementary Figure 6), low certainty of evidence (Supplementary Table 9).

###### 3.1.5.1.2 Synbiotics

The meta-analysis did not yield a statistically significant effect (OR 0.71, 95% CI: 0.33 to 1.55), moderate certainty of evidence (Supplementary Table 9).

###### 3.1.5.1.3 Protein integration

The meta-analysis on protein integration showed no effect in preventing pulmonary infections (OR 0.91, 95% CI: 0.18 to 4.64), very low certainty of evidence (Supplementary Table 9). However, this finding is based on only three studies, as suggested by the wide CI and moderate to high heterogeneity (*I*^2^ = 69.43%).

No analytical studies evaluated the effect of nutritional intervention on pulmonary infections.

#### 3.1.6 LOS

##### 3.1.6.1 Randomized clinical trials

Seventeen studies examined the distribution of nutritional interventions on LOS: 5 studies (17, 18, 21, 33, 35) examined immunonutrition, 4 studies (24, 25, 30, 36) probiotics, 6 studies (23, 26, 28, 31, 32, 37) synbiotics, and 2 studies (20, 22) protein integration.

###### 3.1.6.1.1 Immunonutrition

Immunonutrition was associated with a mean reduction of 2.64 days in LOS (MD −2.64, 95% CI: −5.24 to −0.03, Supplementary Figure 7), though the studies showed high heterogeneity (*I*^2^ 85.74%) and low certainty of evidence (Supplementary Table 10).

###### 3.1.6.1.2 Probiotics

The meta-analysis showed that administration of probiotics was not a reducing factor of LOS (MD −0.94, 95% CI: −2.07 to 0.20), with moderate-to-high heterogeneity (*I*^2^ 69.72%). Subgroup analysis of post-operative LOS confirmed no reduction (MD −0.58, 95% CI: −1.97 to 0.81), low certainty of evidence (Supplementary Table 10).

###### 3.1.6.1.3 Synbiotics

Synbiotics significantly reduced LOS by 9.25 days (MD −9.25, 95% CI: −11.02 to −7.47; *I*^2^ 0.00%, Supplementary Figure 8). However, subgroup analysis of post-operative LOS showed no significant effect (MD −4.05, 95% CI: −14.01 to 5.91), moderate certainty of evidence (Supplementary Table 10).

##### 3.1.6.2 Analytical studies

The analysis included 5 analytical studies (46–48, 53, 54), all of which investigated immunonutrition interventions.

###### 3.1.6.2.1 Immunonutrition

The meta-analysis found that immunonutrition did not influence LOS (MD 1.21, 95% CI: −6.31 to 8.55), with a high heterogeneity among the studies (*I*^2^ 93.33%).

### 3.2 Adverse events

Of the 39 included studies, 23 (12 RCTs and 11 analytical studies) did not clearly report on the adverse events of the interventions (21, 25, 27–29, 32, 35, 37, 39–45, 47, 49–55). Nine studies (all RCTs) reported no therapy-related adverse effects (18, 20, 22–24, 30, 31, 34, 36). The seven studies (five RCTs, two on immunonutrition (17, 33), two on synbiotics (19, 26), one on protein supplementation (38), and two analytical studies (46, 48) on immunonutrition) that reported adverse events highlighted the gastrointestinal side effects of the therapies. The most common symptoms were diarrhea, nausea, and vomiting, even though there were usually non-significant differences between the intervention and control groups.

## 4 Discussion

HAIs impose a substantial public health burden, accounting for significant mortality, morbidity, and healthcare expenditure globally. Postoperative infections, particularly SSIs, represent a preventable component of this burden. SSIs affect approximately 2%–5% of surgical patients in high-income settings and substantially higher proportions in resource-limited contexts, with each infection adding an estimated 7–10 additional hospital days and 3,000–10,000 dollars in direct costs (56, 57). Nutritional interventions offer a potentially scalable, low-risk adjunctive strategy to reduce this burden, yet their integration into routine clinical practice remains inconsistent.

Our analyses revealed substantial heterogeneity in several comparisons, which warrants careful interpretation. This heterogeneity stems from two main sources: (1) Clinical heterogeneity, including variations in nutritional formulations (presence/absence of specific components such as L-arginine, omega-3 fatty acids, RNA nucleotides, PHGG), dosing regimens, timing of administration (preoperative only vs. perioperative), and diverse surgical populations (gastrointestinal, urological, cardiac, orthopedic surgery); (2) Methodological heterogeneity, including differences in study design quality, outcome definitions and surveillance methods, and follow-up duration. The high *I*^2^ values for LOS analyses (85.74% for immunonutrition RCTs, 93.33% for analytical studies) particularly reflect heterogeneity in surgical complexity, baseline patient characteristics, and healthcare system practices. For probiotics and synbiotics, heterogeneity is further compounded by vast differences in bacterial strains, colony-forming units, and probiotic combinations used across studies.

Despite these limitations, our meta-analysis of 39 studies suggest that perioperative nutritional interventions can reduce infectious complications, with immunonutrition demonstrating the most robust effects. Immunonutrition reduced infectious complications by 64% in randomized trials and SSIs by 65%. To illustrate the potential population-level impact, we provide a hypothetical scenario: in a typical 500-bed hospital performing 2,000 major oncological surgeries annually with a baseline SSI rate of 20%, immunonutrition (assuming the pooled OR of 0.35) could theoretically prevent approximately 260 SSIs. Based on published estimates of SSI-associated costs and LOS (58), this could theoretically liberate 1,800–2,600 bed-days and generate estimated savings of 780,000–2,600,000 dollars annually. However, these projections are illustrative estimates based on extrapolations from our pooled effect sizes and published cost data, not empirically derived from our meta-analysis. Actual implementation outcomes would depend on baseline infection rates, patient case mix, and local cost structures.

Probiotics showed moderate effects on specific infections, reducing SSIs by 39%, pneumonia by 58%, and UTIs by 68%, though with lower certainty evidence. Their advantage lies in lower cost, simpler administration, and broader applicability across surgical populations. From a health systems perspective, probiotics may represent a more feasible first-line intervention in resource-constrained settings or for lower-risk procedures where the cost-benefit ratio of immunonutrition is less favorable. Protein supplementation showed statistically significant SSI reduction (OR 0.36), but this finding is based on very low certainty evidence from only four studies with substantial risk of bias, precluding clinical recommendations until large, high-quality RCTs provide robust validation (59).

Implementation feasibility differs substantially across interventions. Immunonutrition requires modifications to preoperative protocols, procurement of specialized formulations, patient education, and compliance monitoring. The predominance of oral administration in effective protocols facilitates outpatient implementation, reducing logistical barriers. However, significant heterogeneity in formulations (presence or absence of L-arginine, omega-3 fatty acids, RNA nucleotides, PHGG), dosing, and timing limits standardization. Our subgroup analyses indicate that products containing L-arginine, omega-3 fatty acids, RNA nucleotides, and soluble fiber demonstrate superior efficacy, suggesting these should be prioritized in protocol development.

The timing of administration emerged as critical, with preoperative supplementation showing stronger effects than combined peri/postoperative regimens. This finding has important practical implications: preoperative-only protocols are simpler to implement, reduce costs, and improve compliance. Healthcare systems should prioritize establishing preoperative immunonutrition pathways for high-risk procedures, particularly in oncological surgery where evidence is strongest and baseline infection rates justify intervention costs.

Critical implementation barriers include lack of standardized protocols, variable adherence to evidence-based formulations, insufficient integration into enhanced recovery pathways, and limited monitoring systems. Addressing this requires development of national or regional clinical guidelines specifying formulation criteria, dosing regimens, target populations, and quality indicators.

Cost-effectiveness analyses are essential for informing resource allocation decisions. Immunonutrition products cost approximately 100–150 dollars per patient course, while preventing one SSI saves 3,000–10,000 dollars in direct costs alone. With a number-needed-to-treat of approximately 8 for SSI prevention, the intervention generates positive returns even before considering indirect costs, antimicrobial stewardship benefits, or patient quality of life.

Most included studies originated from high-income settings in Asia (43.6%) and Europe (33.3%), with limited representation from low- and middle-income countries where HAI burden is highest (57). The interventions’ feasibility and effectiveness may differ substantially in resource-limited settings with higher baseline infection rates, different surgical case mixes, and limited access to specialized nutrition products. Probiotics, with lower costs and simpler supply chains, may offer more equitable solutions in these contexts. Research specifically addressing implementation and effectiveness in diverse healthcare settings is urgently needed to ensure interventions reduce rather than exacerbate global health inequities.

The discordance between RCT and analytical study findings for immunonutrition’s effect on SSIs (RCTs: OR 0.35, 95% CI 0.17–0.71; analytical studies: OR 0.58, 95% CI 0.31–1.07) is clinically important. This likely reflects residual confounding in observational studies, where sicker patients or those at higher baseline risk may be preferentially selected for immunonutrition, partially masking the intervention’s true effect. Differences in patient populations, implementation fidelity, and real-world adherence may also contribute. Critically, this discordance underscores that practice-changing recommendations must be based primarily on RCT evidence, which provides more reliable causal inference through randomization and blinding. Pragmatic cluster-randomized trials evaluating system-level immunonutrition implementation would provide crucial evidence on effectiveness outside controlled research settings and inform scalability assessments.

Our findings have direct implications for infection prevention strategies. Integrating immunonutrition into surgical safety checklists and care bundles for appropriate populations could enhance prevention efforts without competing with existing strategies. This aligns with multimodal infection prevention approaches increasingly recognized as most effective (60).

It is essential to emphasize that nutritional interventions are adjunctive strategies that complement, rather than replace, standard infection prevention measures. Core interventions such as appropriate surgical antibiotic prophylaxis, strict aseptic technique, optimal glycemic control, normothermia maintenance, and standardized surgical site preparation remain foundational. Nutritional supplementation should be integrated into comprehensive, multimodal prevention bundles rather than implemented in isolation.

Several methodological limitations affect evidence certainty. High heterogeneity in multiple analyses (immunonutrition effect on LOS, probiotics on LOS) reflects variability in formulations, populations, and outcome definitions. Small sample sizes in individual studies limit precision, particularly for less common outcomes like BSIs. Publication bias assessment was precluded by insufficient study numbers. The predominance of oncological surgery limits generalizability to other surgical populations. Most studies evaluated short-term outcomes; longer-term impacts on antimicrobial resistance, healthcare utilization, and functional recovery remain unexamined. Importantly, heterogeneity in infection definitions and surveillance methods across studies limits the precision of our pooled estimates. SSIs, BSIs, UTIs, and pneumonia may have been defined and detected using different criteria (e.g., CDC/NHSN definitions vs. institutional protocols), potentially introducing misclassification bias and reducing comparability. Future studies should adopt standardized, internationally recognized outcome definitions to improve evidence synthesis.

From a public health surveillance perspective, current HAI monitoring systems inadequately capture nutritional intervention use, limiting assessment of real-world implementation and effectiveness. Enhancing surveillance to include documentation of perioperative nutritional interventions would enable post-market effectiveness studies, identify implementation gaps, and support quality improvement initiatives.

The moderate-to-low certainty of evidence for several interventions highlights that while current evidence supports implementation of immunonutrition for SSI prevention in high-risk surgery, broader application requires additional research. Implementation should proceed for well-supported interventions while simultaneously generating real-world evidence to refine approaches.

## 5 Conclusion

### 5.1 Response to research question

This systematic review and meta-analysis confirms that perioperative nutritional interventions reduce postoperative infectious complications in surgical patients, with evidence strength varying by intervention type and outcome. Immunonutrition shows the strongest evidence base. RCT data demonstrate robust efficacy in preventing SSIs (OR 0.35, 95% CI 0.17–0.71, moderate certainty evidence) and reducing overall infectious complications (OR 0.36, 95% CI 0.21–0.62, low certainty evidence) in surgical oncology patients. This represents clinically meaningful risk reduction with moderate confidence in the effect estimate.

Probiotics demonstrate protective effects across multiple infection types: SSIs (OR 0.61, low certainty), pneumonia (OR 0.42, low certainty), UTIs (OR 0.32, low certainty), and BSIs (OR 0.42, low certainty). However, evidence certainty is consistently lower than for immunonutrition, reflecting greater heterogeneity in study designs and probiotic formulations.

Synbiotics showed non-significant trends toward benefit across outcomes, with insufficient evidence to support clinical recommendations.

Protein supplementation demonstrated statistical significance for SSI reduction (OR 0.36, 95% CI 0.17–0.75) but carries very low certainty evidence based on only four studies with substantial methodological limitations. This precludes any clinical recommendations until large, well-designed RCTs validate these preliminary findings.

Critically, analytical studies consistently showed weaker and often non-significant effects compared to RCTs. For immunonutrition’s effect on SSIs, observational studies yielded OR 0.58 (95% CI 0.31–1.07) versus RCT evidence of OR 0.35 (95% CI 0.17–0.71). This discordance underscores that practice-changing recommendations must be based primarily on randomized evidence, which provides more reliable causal inference through randomization and blinding.

### 5.2 Clinical implications

For clinical practice, immunonutrition in high-risk oncological surgery represents an evidence-based intervention ready for implementation as standard of care, based on RCT evidence of moderate certainty. Healthcare systems should prioritize establishing preoperative immunonutrition pathways for appropriate surgical populations, integrated within comprehensive infection prevention bundles alongside established measures (antibiotic prophylaxis, aseptic technique, glycemic control).

Probiotics may serve as complementary interventions, particularly in settings where immunonutrition faces implementation barriers or for lower-risk procedures, though their effects are supported by lower certainty evidence. Protein supplementation shows promise but cannot be recommended for infection prevention based on current very low certainty evidence.

### 5.3 Policy and implementation recommendations

National health authorities and surgical societies should establish standardized immunonutrition protocols specifying evidence-based formulations (particularly those containing L-arginine, omega-3 fatty acids, RNA nucleotides, and soluble fiber), preoperative administration timing (based on our subgroup analyses showing superior effects), and clearly defined target populations. Integration into enhanced recovery pathways and surgical safety bundles will facilitate systematic adoption. Healthcare administrators should conduct institution-specific cost-effectiveness analyses to prioritize implementation across surgical services, focusing initially on high-risk gastrointestinal and oncological surgery. While our illustrative economic projections suggest potential substantial savings, actual cost-effectiveness will vary by institution and should be empirically evaluated.

Immunonutrition use in eligible surgical populations should be established as a quality indicator and monitored through national surgical and infection surveillance systems to ensure implementation fidelity and enable ongoing effectiveness evaluation in real-word settings.

### 5.4 Research recommendations

Research priorities must address critical evidence gaps. Large pragmatic multicenter RCT evaluating system-level implementation strategies, real-world effectiveness, and cost-effectiveness across diverse healthcare settings are urgent needed. Studies specifically addressing implementation in low- and middle- income countries are essential to ensure equitable access and avoid exacerbating global health inequities.

For protein supplementation, large well-designed RCTs are prerequisite before any clinical recommendations can be made, given current very low certainty evidence. For probiotics and synbiotics, research must focus on standardizing formulations, dosing, and administration protocols to reduce heterogeneity.

Future studies must adopt standardized, internationally recognized definitions for postoperative infections (SSIs, BSIs, UTIs, pneumonia) to improve comparability and enable more precise meta-analyses.

Long-term outcomes including antimicrobial resistance patterns, healthcare utilization, functional recovery, and patient-reported outcomes should be incorporated. Economic evaluations must accompany effectiveness studies to inform resource allocation decisions.

Enhancing HAI surveillance systems to systematically capture nutritional intervention use (such as national registries linking surgical procedures, nutritional interventions, and infection) will enable post-implementation effectiveness monitoring and identify optimization opportunities.

## Data Availability

The raw data supporting the conclusions of this article will be made available by the authors, without undue reservation.

## Footnotes

1
https://www.gradepro.org/
