# Supplementary material for: The role of nutritional supplementation in preventing postoperative complications: a systematic review and meta-analysis
Source: Front Nutr. 2026 Feb 19;13:1744249. doi: 10.3389/fnut.2026.1744249 (PMC12960083; doi:10.3389/fnut.2026.1744249)

Supplementary Material

# Supplementary Tables

**Table 1. Characteristics of studies included in meta-analysis.**

| **References** | **Study Design, Setting** | **Surgery** | **Intervention** | **Oncological surgery** | **Intervention Group** | | | **Control Group** | | | **Outcomes (relevant for this meta-analysis)** |
| --- | --- | --- | --- | --- | --- | --- | --- | --- | --- | --- | --- |
|  |  |  |  |  | **No.** | **Age** | **Sex, male** | **No.** | **Age** | **Sex, male** |  |
| Braga M. et al., 1999 [12] | Double-Blind RCT, Italy | Laparotomic abdominal surgery | Immunonutrition  (IMPACT ^®^ 1000ml/die x 7 days preoperatively)  *Oral route* | Y | 85 | 60.9 ± 11.9 | 50 (58.8) | 86 | 60.8 ± 9.7 | 56 (65.1) | Infectious complication, SSI, BSI, UTI, Pneumonia, LOS |
| Senkal M. et al., 1999 [13] | Double-Blind RCT, Germany | Laparotomic abdominal surgery | Immunonutrition  (IMPACT ^®^ 1000ml/die x at least 5 days preoperatively)  *Oral route* | Y | 89 | 64.0 ± 11.0 | 48 (53.9) | 89 | 67.0 ± 9.0 | 52 (58.4) | Infectious complication, LOS |
| Anderson A. D. G. et al., 2003 [14] | Double-Blind RCT, UK | Laparotomic abdominal surgery | Synbiotic (Trevis^®^  3 cps/die and 16 g  oligofructose powder x 2/die  x 1–2 weeks preoperatively)  *Oral route* | Mixed | 72 | 71 [47–76] | 38 (52.8) | 65 | 71 [66–80] | 42 (64.6) | BSI |
| Takeuchi H. et al., 2007 [38] | Retrospective Cohort, Japan | Thoracic surgery | Immunonutrition (IMPACT ^®^ 250-1500Kcal/die x 5 days, preoperatively)  *Enteral route* | Y | 6 | 61.5 ± 5.0 | 5 (83.3) | 20 | 64.6 ± 7.6 | 19 (95.0) | BSI, SSI |
|  | Retrospective Cohort, Japan | Thoracic surgery | Immunonutrition (IMPACT ^®^ 250-1500Kcal/die x 5 days preoperatively and 1500 Kcal/die postoperatively)  *Enteral route* | Y | 14 | 65.0 ± 7.4 | 13 (92.9) | 20 | 64.6 ± 7.6 | 19 (95.0) | BSI, SSI |
| Okamoto Y. et al., 2009 [16] | RCT, Japan | Laparotomic abdominal surgery | Immunonutrition (IMPACT ^®^ 750ml/die x 7 days preoperatively)  *Oral route* | Y | 30 | 66.9 ± 11.5 | 20 (66.7) | 30 | 70.9 ± 13.2 | 22 (73.3) | Infectious complication, SSI, LOS |

| **References** | **Study Design, Setting** | **Surgery** | **Intervention** | **Oncological surgery** | **Intervention Group** | | | **Control Group** | | | **Outcomes (relevant for this meta-analysis)** |
| --- | --- | --- | --- | --- | --- | --- | --- | --- | --- | --- | --- |
|  |  |  |  |  | **No.** | **Age** | **Sex, male** | **No.** | **Age** | **Sex, male** |  |
| De Luis D. A. et al., 2010 [17] | Double-Blind RCT, Spain | ENT surgery | Protein integration (Arginin 20g/die x at least 20 days postoperatively)  *Enteral route* | Y | 58 | 62.5 ± 13.6 | 45 (77.6) | 57 | 62.6 ± 10.4 | 45 (78.9) | LOS |
| Liu Z. et al., 2011 [19] | Double-Blind RCT, China | Laparotomic abdominal surgery | Probiotic (encapsulated  bacteria containing Lactobacillus plantarum ≥10^11^ CFU⁄ g, Lactobacillus acidophilus ≥7 X10^10^ CFU⁄ g and Bifido-bacterium longum ≥5 X10^10^ CFU ⁄ g, 2 g ⁄ die, in a total daily dose of 2.6 X 10^14^ CFU for 6 days preoperatively and 10 days postoperatively)  *Oral route* | Y | 50 | 65.3 ± 11.0 | 28 (56.0) | 50 | 65.7 ± 9.9 | 31 (62.0) | SSI, BSI, UTI, Pneumonia, LOS |
| Eguchi S. et al., 2011 [18] | RCT, Japan | Liver transplantation | Synbiotic (Yakult BL antiflatulent^®^  and galactooligosaccharides 15 g/d, Oligomate 55^®^ x 3/die from 2 days preoperatively for 2 weeks postoperatively)  *Oral or enteral route* | Mixed | 25 | 56 [33–66] | 13 (52.0) | 25 | 57 [25–68] | 16 (64.0) | Infectious complication, LOS |
| Estívariz C. F. et al., 2011 [15] | Double-Blind RCT, Georgia | Abdominal, cardiac, vascular surgeries | Protein integration (Clinisol®15%, 1.5 g/kg/die)  *Parenteral route* | N | 15 | 51.0 ± 3.0 | 10 (66.7) | 17 | 51.0 ± 3.0 | 14 (82.4) | SSI, BSI, UTI, Pneumonia |
|  | Double-Blind RCT, Georgia | Abdominal, cardiac, vascular surgeries | Protein integration  (Clinisol®15%, 1 g/kg/die and Dipeptiven® 0,5g/kg/die)  *Parenteral route* | N | 15 | 61.0 ± 4.0 | 9 (60.0) | 12 | 67.0 ± 3.0 | 7 (58.3) | SSI, BSI, UTI, Pneumonia, LOS |
| Kaido T. et al., 2012 [39] | Retrospective Cohort, Japan | Liver transplantation | Immunonutrition (MHN-  02, MEIN^®^, 20-40 ml/die, 10-14 day postoperatively)  *Enteral route* | Mixed | 40 | 47.8 ± 14.8 | 19 (47.5) | 36 | 53.2 ± 13.4 | 14 (38.9) | BSI |
| Zhang Ji-Wei et al., 2012 [20] | Double-Blind RCT, China | Laparotomic abdominal surgery | Probiotic (3 oral capsules containing 0.21 g (108 cfu/g) of B longum, L acidophilus and Enterococcus faecalis, x 3/die, from day -5 to day -3 preoperatively)  *Oral route* | Y | 30 | 67.5  [45.0–87.0] | 10 (33.3) | 30 | 61.5  [46.0–82.0] | 14 (46.7) | Infectious complication, SSI, BSI, Pneumonia, LOS |
| Bertrand N. et al., 2013 [40] | Case-Control, France | Cistectomy | Immunonutrition (Oral Impact^®^ x 3/die for 7 days preoperatively)  *Oral route* | Y | 30 | 69.6 [52–85] | 23 (76.7) | 30 | 68.9 [50–89] | 25 (83.3) | Infectious complication |

| **References** | **Study Design, Setting** | **Surgery** | **Intervention** | **Oncological surgery** | **Intervention Group** | | | **Control Group** | | | **Outcomes (relevant for this meta-analysis)** |
| --- | --- | --- | --- | --- | --- | --- | --- | --- | --- | --- | --- |
|  |  |  |  |  | **No.** | **Age** | **Sex, male** | **No.** | **Age** | **Sex, male** |  |
| Sadahiro S. et al., 2013 [22] | RCT, Japan | Laparotomic (72%) and laparoscopic (28%) abdominal surgery | Probiotic (Biofermin^®^ 3 tablets x 3/die, for 7 days preoperatively and from day 5 to day 10 postoperatively)  *Oral route* | Y | 100 | 67.0 ± 9.0 | 49 (49.0) | 95 | 66.0 ± 12.0 | 51 (53.7) | Infectious complication, SSI |
| Russolillo N. et al., 2014 [21] | RCT, Italy | Elective extrahepatic bile duct resection | Synbiotic (Prebiotic^®^ 1 sachet x 2/die for at least 1 week preoperatively, and as tolerated postoperatively until discharge)  *Oral route* | Y | 20 | 63.0  [46.0–78.0] | 14 (70.0) | 20 | 63.5  [34.0–77.0] | 10 (50.0) | Infectious complication, BSI, Pneumonia, LOS |
| Yokoyama Y. et al., 2014 [23] | RCT, Japan | Laparotomic abdominal surgery | Synbiotic (one 80-ml bottle of Yakult^®^ 400 and one  100-ml bottle of MILMIL-S^®^ x 1/die, for 7 days preoperatively)  *Oral or enteral route* | Y | 21 | 65 [53–77] | 19 (90.5) | 21 | 66 [25–77] | 18 (85.7) | Infectious complication, SSI, BSI, Pneumonia, LOS |
| Kotzampassi K. et al., 2015 [24] | Double-Blind RCT, Greece | Laparotomic abdominal surgery | Probiotic (LactoLevure^®^ 1 cps x 2/die, from 1 day before surgery to 14 days postoperatively  *Oral route* | Y | 84 | 65.9 ± 11.5 | 57 (67.9) | 80 | 66.4 ± 11.9 | 58 (51.5) | SSI, UTI, Pneumonia |
| Liu Z. et al., 2015 [25] | Double-Blind RCT, China | Laparotomic abdominal surgery | Probiotic (2 g/die of a combination of LP ≥10^11^ cfu/g, LA-11 ≥7.0 × 10^10^ cfu/g and BL-88 ≥5.0 × 10^10^ cfu/g, for 6 days preoperatively and 10 days postoperatively)  *Oral route* | Y | 66 | 65.6 ± 18.2 | 35 (53.0) | 68 | 60.2 ± 16.2 | 35 (51.5) | SSI, UTI, Pneumonia, LOS |
| Rammohan A. et al., 2015 [26] | Single-Blind RCT, India | Pancreatic surgery | Synbiotic (Bifiliac HP^®^ 1 tablet x 3/die for 5 days preoperatively and 10 day postoperatively)  *Oral route* | N | 39 | 43.2 ± 9.2 | 25 (64.1) | 36 | 43.4 ± 8.7 | 23 (63.9) | SSI, BSI, UTI, Pneumonia, LOS |
| Sommacal H. M. et al., 2015 [27] | Double-Blind RCT, Brazil | Laparotomic abdominal surgery | Synbiotic (1 capsule  containing Lactobacillus acidophilus 10,1 x10^9^ CFU, Lactobacillus rhamnosus HS 111,1 x10^9^ CFU, Lactobacillus casei 10,1x10^9^ CFU, Bifidobacterium bifidum, 1 x10^9^ CFU, and fructooligosaccharides 100 mg, x2/die, for four days preoperatively and 10 days postoperatively)  *Oral route* | Y | 23 | 56 [45–80] | NA | 23 | 63 [44–85] | NA | Infectious complication, LOS |

| **References** | **Study Design, Setting** | **Surgery** | **Intervention** | **Oncological surgery** | **Intervention Group** | | | **Control Group** | | | **Outcomes (relevant for this meta-analysis)** |
| --- | --- | --- | --- | --- | --- | --- | --- | --- | --- | --- | --- |
|  |  |  |  |  | **No.** | **Age** | **Sex, male** | **No.** | **Age** | **Sex, male** |  |
| Hamilton-Reeves J. M. et al., 2016 [28] | RCT, USA | Cistectomy | Immunonutrition (Impact Advanced Recovery^®^ 3 cartons/die, for 5 days preoperatively and 5 days postoperatively)  *Oral route* | Y | 14 | NA | NA | 15 | NA | NA | Infectious complication, LOS |
| Komatsu S. et al., 2016 [29] | RCT, Japan | Laparotomic abdominal surgery | Synbiotic (one 80-ml bottle of Yakult Ace^®^ and and one 100-ml bottle of MILMIL-S^®^ x1/die, for 7-11 days preoperatively and reintroduced 2-7 days postoperatively)  *Oral route* | Y | 168 | 69 [29–92] | 92 (54.8) | 194 | 69 [30–89] | 118 (60.8) | Infectious complication, SSI |
| Moya P. et al., 2016 [30] | Single-Blind RCT, Spain | Laparotomic (24%) and laparoscopic (75%) abdominal surgery | Immunonutrition (ATEMPERO^®^, 400 ml/die for 7 days preoperatively and for 5 days postoperatively)  *Oral route* | Y | 122 | 70 [42–88] | 62 (50.8) | 122 | 68 [41–89] | 69 (56.6) | SSI, LOS |
| Silvestri S. et al., 2016 [41] | Prospective Cohort, Italy | Laparotomic abdominal surgery | Immunonutrition (Oral Impact^®^ x 3/die for at least 5 days preoperatively)  *Oral route* | Y | 48 | 62.3 ± 11.5 | 26 (54.2) | 48 | 63.9 ± 11.8 | 20 (41.7) | Infectious complication, SSI, LOS |
| Yang Y. et al., 2016 [31] | Double-Blind RCT, China | Laparotomic abdominal surgery | Probiotic (Bifico^®^ cps for 5 days preoperatively and for 7 days postoperatively)  *Oral or enteral route* | Y | 30 | 63.9 ± 12.3 | 15 (50.0) | 30 | 62.2 ± 11.1 | 12 (40.0) | SSI, UTI, Pneumonia, LOS |
| Yokoyama Y. et al., 2016 [32] | RCT, Japan | Pancreatic surgery | Synbiotic (one 80-ml bottle of Yakult 400^®^ and and one 100-ml bottle of MILMIL-S^®^ x1/die, for 7 days preoperatively)  *Oral route* | Y | 22 | 65 [54–82] | 6 (27.3) | 22 | 65 [41–83] | 6 (27.3) | SSI, Pneumonia, LOS |
| Burden S. T. et al., 2017 [33] | Single-Blind RCT, UK | Laparoscopic (66%) + Laparotomic surgery (34%) abdominal | Protein integration (Fortisip  Compact^®^ 250 ml/die, for at least 5 days preoperatively)  *Oral route* | Y | 55 | 70.5 ± 11.7 | 35 (63.6) | 46 | 68.9 ± 11.5 | 32 (69.6) | Infectious complication, SSI, UTI, Pneumonia |
| Flesch A. T. et al., 2017 [34] | Double-Blind RCT, Brazil | Laparotomic (87%) and laparoscopic (13%) abdominal surgery | Synbiotic (2 sachets/die containing Lacto bacillus acidophilus NCFM 10^9^, Lactobacillus rham-  nosus HN001 10^9^, Lactobacillus paracasei LPC-37 10^9^, Bifidobacterium lactis HN019 10^9^ and fructooligosaccharides 6g, for 5 days preoperatively and for 14 days postoperatively)  *Oral route* | Y | 49 | 64.5 ± 11.4 | 18 (36.7) | 42 | 61.1 ± 13.4 | 19 (45.2) | SSI, Pneumonia |

| **References** | **Study Design, Setting** | **Surgery** | **Intervention** | **Oncological surgery** | **Intervention Group** | | | **Control Group** | | | **Outcomes (relevant for this meta-analysis)** |
| --- | --- | --- | --- | --- | --- | --- | --- | --- | --- | --- | --- |
|  |  |  |  |  | **No.** | **Age** | **Sex, male** | **No.** | **Age** | **Sex, male** |  |
| Martin II R. C. G. et al., 2017 [42] | Prospective Cohort, USA | Pancreatic surgery | Immunonutrition (IMPACT ADVANCED RECOVERY^®^, 3/die for 5 days preoperatively)  *Oral route* | Y | 27 | 60 [27–81] | 12 (44.4) | 44 | 62 [47–75] | 30 (68.2) | Infectious complication, SSI, LOS |
| Hertlein L. et al., 2018 [43] | Prospective Cohort, Germany | Laparotomic ovarian surgery | Immunonutrition (IMPACT^®^ 3/die, for 5 days preoperatively and at least 5 days postoperatively  *Oral route* | Y | 28 | 70 [51–81] | NA | 19 | 68 [36–77] | NA | Infectious complication, SSI, BSI, LOS |
| Kamo N. et al., 2018 [44] | Retrospective Cohort, Japan | Liver transplantation | Immunonutrition (MHN-  02, MEIN^®^, initial rate 20 ml/h from the first day postoperatively)  *Enteral route* | Mixed | 164 | 54 [18–69] | 86 (52.4) | 115 | 55 [18–69] | 51 (44.3) | BSI |
| Feguri G. R. et al., 2019 [35] | Double-Blind RCT, Brazil | Coronary artery bypass | Immunonutrition (200 ml of maltodextrin 12.5%, two hours before surgery)  *Parenteral route* | N | 14 | 60.9 ± 10.6 | 12 (85.7) | 14 | 63.4 ± 8.6 | 10 (71.4) | Infection complications |
| Claudino M. M. et al., 2020 [45] | Retrospective Cohort, Brazil | Laparotomic abdominal surgery | Immunonutrition  (polymeric, hyperprotein diet, enriched with arginine, omega-3 fatty acids, and  nucleotides, totaling 600 mL and 600 kcal/die, for 5 to 7 days preoperatively)  *Oral or enteral route* | Y | 56 | 63.7 ± 11.8 | 32 (57.1) | 108 | 61.1 ± 13.0 | 56 (51.9) | SSI |
| Furukawa A. et al., 2020 [46] | Retrospective Cohort, Japan | Pancreatic surgery | Immunonutrition (Oral Impact^®^ 1000kcal/die for 5 days preoperatively)  *Oral route* | Y | 19 | NA | NA | 55 | NA | NA | Infectious complication |
|  | Retrospective Cohort, Japan | Pancreatic surgery | Immunonutrition (Oral Impact^®^ 1000kcal/die for 5 days preoperatively)  *Oral route* | Y | 72 | NA | NA | 152 | NA | NA | Infectious complication |

| **References** | **Study Design, Setting** | **Surgery** | **Intervention** | | **Oncological surgery** | | **Intervention Group** | | | | **Control Group** | | | **Outcomes (relevant for this meta-analysis)** |
| --- | --- | --- | --- | --- | --- | --- | --- | --- | --- | --- | --- | --- | --- | --- |
|  |  |  |  |  |  |  | **No.** | | **Age** | **Sex, male** | **No.** | **Age** | **Sex, male** |  |
| Patel Y. S. et al., 2021 [48] | Retrospective Cohort, USA | Cistectomy | | Immunonutrition (immunonutrition beverage with ω-3, arginine, and nucleotides, calories 200 kcal, protein 18 g 3 times a day for 5 days before surgery and a complex carbohydrate beverage with maltodextrin, calories 200 kcal, protein 0 g the night before surgery and 2 hours prior to surgery)  *Oral route* | | Y | | 78 | 71.0 [43.0–87.0] | 70 (89.7) | 92 | 71.5 [41.0–89.0] | 78 (84.8) | Infectious complication, LOS |
| Ferrero A. et al., 2022 [47] | Prospective Cohort, Italy | Laparotomic ovarian surgery | | Immunonutrition (Oral Impact^®^ 2 servings/die for 5-10 days preoperatively) | | Y | | 42 | 62.7 [40.0–82.0] | NA | 42 | 62.3 [32.0–78.0] | NA | Infectious complication |
| Tzikos G. et al., 2022 [36] | Double-Blind RCT, Switzerland | Neurosurgery, thoracostomies, exploratory laparotomies, orthopedic surgery, and others | | Probiotic (LactoLevure^®^ two sachets twice daily, for 15 days postoperatively)  *Oral and enteral route* | | N | | 53 | 38.4 ± 16.9 | 50 (94.3) | 50 | 44.1 ± 13.9 | 40 (80.0) | SSI |
| Ogilvie Jr. J. et al., 2023 [49] | Prospective Cohort, USA | Laparoscopic (75%) + Laparotomic abdominal surgery (25%) | | Immunonutrition (Ensure Surgery^®^ 3/die for 5 days preoperatively)  *Oral route* | | Mixed | | 514 | 60.6 ± 15.0 | 232 (45.1) | 312 | 59.9 ± 17.1 | 138 (44.2) | SSI, LOS |
| Saleh H. et al., 2023 [37] | RCT, New York | Lumbar spine surgery | | Protein integration (protein shake with 30 g of protein,150 calories, and 1 g of sugar 2/die, postoperatively, during the hospital stay and for 15 days after discharge)  *Oral route* | | N | | 46 | 63.6 | 19 (41.3) | 57 | 63.6 | 22 (38.6) | SSI |
| Takeuchi H. et al., 2024 [38] | Retrospective Cohort Study, Japan | Cistectomy | | Immunonutrition (IMPACT^®^ 4/die, for 5 days preoperatively)  *Oral route* | | Y | | 43 | 72 [70–77] | 31 (72.1) | 43 | 71 [64–76] | 33 (76.7) | Infection complications, SSI |

Mean ± Standard Deviation, Median [Interquartile Range], Mean (Min-Max); NA = not applicable, SSI = Surgical Site Infections, BSI = Bloodstream Infection, UTI = Urinary Tract Infection, LOS = Hospital Length of stay

**Table 2. Characteristics of the study outcomes included in the meta-analysis.**

| **References** | **Infectious complication (n/N, %)** | | **SSI (n/N, %)** | | **BSI (n/N, %)** | | **UTI (n/N, %)** | | **Pneumonia (n/N, %)** | |
| --- | --- | --- | --- | --- | --- | --- | --- | --- | --- | --- |
|  | **Intervention** | **Control** | **Intervention** | **Control** | **Intervention** | **Control** | **Intervention** | **Control** | **Intervention** | **Control** |
| Braga M. et al., 1999 [12] | 9/85  10.59 | 21/86  24.42 | 3/85  3.53 | 6/86  6.98 | 0/85  0.00 | 2/86  2.33 | 2/85  2.35 | 3/86  3.49 | 4/85  4.71 | 9/86  10.47 |
| Senkal M. et al., 1999 [13] | 10/89  11.24 | 18/89  20.22 | NA | NA | NA | NA | NA | NA | NA | NA |
| Anderson A. D. G. et al., 2003 [14] | NA | NA | NA | NA | 23/72  31.94 | 20/65  30.77 | NA | NA | NA | NA |
| Takeuchi H. et al., 2007 [38] | NA | NA | 2/6  33.33 | 6/20  30.00 | 1/6  16.67 | 2/20  10.00 | NA | NA | NA | NA |
|  | NA | NA | 0/14  0.00 | 6/20  30.00 | 0/14  0.00 | 2/20  10.00 | NA | NA | NA | NA |
| Okamoto Y. et al., 2009 [16] | 2/30  6.67 | 8/30  26.67 | 1/30  3.33 | 2/30  6.67 | NA | NA | NA | NA | NA | NA |
| Liu Z. et al., 2011 [19] | NA | NA | 3/50  6.00 | 5/50  10.00 | 1/50  2.00 | 7/50  14.00 | 1/50  2.00 | 6/50  12.00 | 2/50  4.00 | 5/50  10.00 |
| Eguchi S. et al., 2011 [18] | 1/25  4.00 | 6/25  24.00 | NA | NA | NA | NA | NA | NA | NA | NA |
| Estívariz C. F. et al., 2011 [15] | NA | NA | 1/15  6.67 | 1/17  5.88 | 4/15  26.67 | 5/17  29.41 | 3/15  20.00 | 4/17  23.53 | 7/15  46.67 | 4/17  23.53 |
|  | NA | NA | 1/15  6.67 | 1/12  8.33 | 0/15  0.00 | 5/12  41.67 | 3/15  20.00 | 5/12  41.67 | 5/15  33.33 | 9/12  75.00 |

| **References** | **Infectious complication (n/N, %)** | | | | | | **SSI (n/N, %)** | | | | | | | | | | | **BSI (n/N, %)** | | | | | | | | | | | | | | **UTI (n/N, %)** | | | | | | | | | | | | | | **Pneumonia (n/N, %)** | | | | | | |
| --- | --- | --- | --- | --- | --- | --- | --- | --- | --- | --- | --- | --- | --- | --- | --- | --- | --- | --- | --- | --- | --- | --- | --- | --- | --- | --- | --- | --- | --- | --- | --- | --- | --- | --- | --- | --- | --- | --- | --- | --- | --- | --- | --- | --- | --- | --- | --- | --- | --- | --- | --- | --- |
|  | **Intervention** | | **Control** | | | | **Intervention** | | | | | | **Control** | | | | | **Intervention** | | | | | | | **Control** | | | | | | | **Intervention** | | | | | | | **Control** | | | | | | | **Intervention** | | | | | | **Control** |
| Kaido T. et al., 2012 [39] | NA | | NA | | | NA | | | | | NA | | | | | 6/40  15.00 | | | | | | | | 17/36  47.22 | | | | | | | | NA | | | | | | NA | | | | | | | NA | | | | | | | NA |
| Zhang Ji-Wei et al., 2012 [20] | 3/30  10.00 | | 10/30  33.33 | | | 1/30  3.33 | | | | | 4/30  13.33 | | | | | 2/30  6.67 | | | | | | | | 9/30  30.00 | | | | | | | | NA | | | | | | NA | | | | | | | 1/30  3.33 | | | | | | | 4/30  13.33 |
| Bertrand N. et al., 2013 [40] | 7/30  23.33 | | 18/30  60.00 | | | NA | | | | | NA | | | | | NA | | | | | | | | NA | | | | | | | | NA | | | | | | NA | | | | | | | NA | | | | | | | NA |
| Sadahiro S. et al., 2013 [22] | 24/100  24.00 | | 24/95  25.26 | | | 22/100  22.00 | | | | | 22/95  23.16 | | | | | NA | | | | | | | | NA | | | | | | | | NA | | | | | | NA | | | | | | | NA | | | | | | | NA |
| Russolillo N. et al., 2014 [21] | 5/20  25.00 | | 10/20  50.00 | | | NA | | | | | NA | | | | | 5/20  25.00 | | | | | | | | 7/20  35.00 | | | | NA | | | | | | | | NA | | | | | 3/20  15.00 | | | | | | | | | 4/20  20.00 | | |
| Yokoyama Y. et al., 2014 [32] | 9/21  42.86 | | 6/21  28.57 | | | 2/21  9.52 | | | | | 1/21  4.76 | | | | | 2/21  9.52 | | | | | | | | 0/21  0.00 | | | | NA | | | | | | | | NA | | | | | 7/21  33.33 | | | | | | | | | 5/21  23.81 | | |
| Kotzampassi K. et al., 2015 [24] | NA | | NA | | | 6/84  7.14 | | | | | 16/80  20.00 | | | | | 6/84  7.14 | | | | | | | | 8/80  10.00 | | | | 4/84  4.76 | | | | | | | | 6/80  7.50 | | | | | 2/84  2.38 | | | | | | | | | 9/80  11.25 | | |
| Liu Z. et al., 2015 [25] | NA | | NA | | | 6/66  9.09 | | | | | 8/68  11.76 | | | | | 7/66  10.61 | | | | | | | | 6/68  8.82 | | | | 1/66  1.52 | | | | | | | | 9/68  13.24 | | | | | 6/66  9.09 | | | | | | | | | 8/68  11.76 | | |
| Rammohan A. et al., 2015 [26] | NA | | NA | | | 3/39  2.56 | | | | | 8/36  22.22 | | | | | 0/39  0.00 | | | | | | | | 1/36  2.78 | | | | 0/39  0.00 | | | | | | | | 2/36  5.56 | | | | | 2/39  5.13 | | | | | | | | | 3/36  5.56 | | |
| Sommacal H. M. et al., 2015 [27] | 6/23  26.10 | | 19/23  82.61 | | | NA | | | | | NA | | | | | NA | | | | | | | | NA | | | | NA | | | | | | | | NA | | | | | NA | | | | | | | | | NA | | |
| Hamilton-Reevesa J. M. et al., 2016 [28] | 1/14  7.14 | | 4/15  26.67 | | | NA | | | | | NA | | | | | NA | | | | | | | | NA | | | | NA | | | | | | | | NA | | | | | NA | | | | | | | | | NA | | |
| **References** | | **Infectious complication (n/N, %)** | | | | | | | | | | **SSI (n/N, %)** | | | | | | | | | | | **BSI (n/N, %)** | | | | | | | | | | | | **UTI (n/N, %)** | | | | | | | | | | | | | **Pneumonia (n/N, %)** | | | | |
|  |  | **Intervention** | | | | | | **Control** | | | | **Intervention** | | | | | **Control** | | | | | | **Intervention** | | | | | | | **Control** | | | | | **Intervention** | | | | | | | | **Control** | | | | | **Intervention** | | | **Control** | |
| Komatsu S. et al., 2016 [29] | 31/168  18.45 | | 49/194  25.26 | | | 14/168  8.33 | | | | | 24/194  12.37 | | | | | NA | | | | | | | | NA | | | | NA | | | | | | | | NA | | | | | NA | | | | | | | | | NA | | |
| Moya P. et al., 2016 [30] | NA | | NA | | | 7/122  5.74 | | | | | 21/122  17.21 | | | | | NA | | | | | | | | NA | | | | NA | | | | | | | | NA | | | | | NA | | | | | | | | | NA | | |
| Silvestri S. et al., 2016 [41] | 11/48  22.92 | | 21/48  43.75 | | | 7/48  14.58 | | | | | 11/48  22.92 | | | | | 0/48  0.00 | | | | | | | | 1/48  2.08 | | | | NA | | | | | | | | NA | | | | | NA | | | | | | | | | NA | | |
| Yang Y. et al., 2016 [31] | NA | | NA | | | 1/30  3.33 | | | | | 1/30  3.33 | | | | | 3/30  10.00 | | | | | | | | 9/30  30.00 | | | | 2/30  6.67 | | | | | | | | 2/30  6.67 | | | | | 3/30  10.00 | | | | | | | | | 5/30  16.67 | | |
| Yokoyama Y. et al., 2016 [32] | NA | | NA | | | 3/22  13.64 | | | | | 2/22  9.09 | | | | | 3/22  13.64 | | | | | | | | 4/22  18.18 | | | | NA | | | | | | | | NA | | | | | 1/22  4.55 | | | | | | | | | 1/22  4.55 | | |
| Burden S. T. et al., 2017 [33] | 17/55  30.91 | | 21/46  45.65 | | | 11/55  20.00 | | | | | 17/46  36.96 | | | | | NA | | | | | | | | NA | | | | 4/55  7.27 | | | | | | | | 6/46  13.04 | | | | | 5/55  9.09 | | | | | | | | | 3/46  6.52 | | |
| Flesch A. T. et al., 2017 [34] | | NA | | NA | | | | | 1/49  2.04 | | | | | 9/42  21.43 | | | | | NA | | NA | | | | | | NA | | | | | | NA | | | | | | | 0/49  0.00 | | | | | | | | | | 4/42  9.52 | | |
| Martin II R. C. G. et al., 2017 [42] | | 2/27  7.41 | | | 10/44  22.73 | | | | | 1/27  3.70 | | | | | 4/44  9.09 | | | | | NA | | | | | | NA | | | | | NA | | | | | | NA | | | | | | | NA | | | | | | NA | | |
| Hertlein L. et al., 2018 [43] | | 6/28  21.43 | | | 5/19  26.32 | | | | | 1/28  3.57 | | | | | 0/19  0.00 | | | | | 0/28  0.00 | | | | | | 1/19  5.26 | | | | | NA | | | | | | NA | | | | | | | NA | | | | | | NA | | |
| Kamo N. et al., 2018 [44] | | NA | | | NA | | | | | NA | | | | | NA | | | | | 40/164  24.39 | | | | | | 48/115  41.74 | | | | | NA | | | | | | NA | | | | | | | NA | | | | | | NA | | |
| Feguri G. R. et al., 2019 [35] | | 1/14  7.14 | | | 2/14  14.29 | | | | | NA | | | | | NA | | | | | NA | | | | | | NA | | | | | NA | | | | | | NA | | | | | | | NA | | | | | | NA | | |
| Claudino M. M. et al., 2020 [45] | | NA | | | NA | | | | | 5/56  8.93 | | | | | 13/108  12.04 | | | | | NA | | | | | | NA | | | | | NA | | | | | | NA | | | | | | | NA | | | | | | NA | | |
| **References** | | **Infectious complication (n/N, %)** | | | | | | | | | | **SSI (n/N, %)** | | | | | | | | | | **BSI (n/N, %)** | | | | | | | | | | | | **UTI (n/N, %)** | | | | | | | | | | | | | **Pneumonia (n/N, %)** | | | | | |
|  |  | **Intervention** | | | | | | **Control** | | | | **Intervention** | | | | | **Control** | | | | | **Intervention** | | | | | | | **Control** | | | | | **Intervention** | | | | | | | | **Control** | | | | | **Intervention** | | **Control** | | | |
| Furukawa A. et al., 2020 [46] | | 5/19  26.32 | | | 46/55  83.64 | | | | | NA | | | | | NA | | | | | NA | | | | | | NA | | | | | NA | | | | | | NA | | | | | | | NA | | | | | | NA | | |
|  |  | 23/72  31.94 | | | 70/152  46.05 | | | | | NA | | | | | NA | | | | | NA | | | | | | NA | | | | | NA | | | | | | NA | | | | | | | NA | | | | | | NA | | |
| Patel Y. S. et al., 2021 [48] | | 33/78  42.31 | | | 34/92  36.96 | | | | | NA | | | | | NA | | | | | NA | | | | | | NA | | | | | NA | | | | | | NA | | | | | | | NA | | | | | | NA | | |
| Ferrero A. et al., 2022 [47] | | 1/42  2.38 | | | 7/42  16.67 | | | | | NA | | | | | NA | | | | | NA | | | | | | NA | | | | | NA | | | | | | NA | | | | | | | NA | | | | | | NA | | |
| Tzikos G. et al., 2022 [36] | | NA | | | NA | | | | | 13/53  24.53 | | | | | 23/50  46.00 | | | | | NA | | | | | | NA | | | | | NA | | | | | | NA | | | | | | | NA | | | | | | NA | | |
| Ogilvie Jr. J. et al., 2023 [49] | | NA | | | NA | | | | | 35/514  6.81 | | | | | 20/312  6.41 | | | | | NA | | | | | | NA | | | | | NA | | | | | | NA | | | | | | | NA | | | | | | NA | | |
| Saleh H. et al., 2023 [37] | | NA | | | NA | | | | | 1/46  2.17 | | | | | 9/57  15.79 | | | | | NA | | | | | | NA | | | | | NA | | | | | | NA | | | | | | | NA | | | | | | NA | | |
| Takeuchi H. et al., 2024 [38] | | 19/43  44.19 | | | 32/43  74.42 | | | | | 2/43  4.65 | | | | | 11/43  25.58 | | | | | NA | | | | | | NA | | | | | NA | | | | | | NA | | | | | | | NA | | | | | | NA | | |

NA = not applicable, SSI = Surgical Site Infections, BSI = Bloodstream Infection, UTI = Urinary Tract Infection.

The study by De Luis D. A. et al., 2010 was not included in the table because it considered only length of stay (LOS) as the outcome.

**Table 3. Impact of Immunonutrition on postoperative infectious complications.**

| **Outcome** | **Subgroup analysis** | | **RCT** | **Analytical** |
| --- | --- | --- | --- | --- |
|  |  |  | **OR [95% CI]**  **(N. Studies)** | **OR [95% CI]**  **(N. Studies)** |
| **Infectious complication** | **All** |  | **0.36 [0.21, 0.62]** (5) | **0.32 [0.17, 0.61]** (9) |
|  | **By Route** | Oral  Oral and/or Enteral/Parenteral | **0.24 [0.07, 0.78]** (3)  **0.41 [0.22, 0.74]** (2) | **0.32 [0.17, 0.61]** (9)  NA |
|  | **By Time** | Preoperative  Preoperative and Intra/Postoperative | **0.25 [0.06, 0.98]** (2)  **0.39 [0.22, 0.70]** (3) | **0.29 [0.14, 0.58]** (8)  NA |
|  | **By Country** | Europe  Asia  North America  South America | **0.41 [0.22, 0.74]** (2)  NA  NA  NA | **0.32 [0.18, 0.58]** (4)  **0.24 [0.08, 0.76]** (3)  0.36 [0.03, 5.03] (2)  NA |
|  | **By Age** | ≤ 65 years  > 65 years | **0.38 [0.17, 0.84]** (2)  NA | **0.23 [0.11, 0.47]** (3)  **0.38 [0.17, 0.85]** (6) |
|  | **By Product** | Impact  Not Reported | **0.36 [0.21, 0.62]** (4)  NA | **0.26 [0.15, 0.47]** (8)  NA |
| **Surgical Site Infection (SSI)** | **All** |  | **0.35 [0.17, 0.71]** (3) | 0.58 [0.31, 1.07] (8) |
|  | **By Route** | Oral  Oral and/or Enteral/Parenteral  Enteral | **0.31 [0.13, 0.72]** (2)  NA  NA | 0.51 [0.21, 1.27] (5)  0.37 [0.05, 2.72] (2)  NA |
|  | **By Time** | Preoperative  Preoperative and Intra/Postoperative  Intra/Postoperative | NA  **0.34 [0.16, 0.72]** (2)  NA | 0.54 [0.26, 1.12] (5)  0.38 [0.01, 9.74] (2)  NA |
|  | **By Country** | Europe  Asia  North America  South America | **0.34 [0.16, 0.72]** (2)  NA  NA  NA | 0.66 [0.25, 1.75] (2)  0.26 [0.05, 1.29] (3)  0.52 [0.08, 3.49] (2)  NA |
|  | **By Age** | ≤ 65 years  > 65 years | NA  NA | 0.75 [0.46, 1.23] (6)  0.37 [0.03, 4.73] (2) |
|  | **By Product** | Impact  Not Reported | 0.49 [0.14, 1.66] (2)  NA | 0.37 [0.16, 0.86] (6)  0.98 [0.59, 1.62] (2) |
| **Bloodstream Infection (BSI)** | **All** |  | NA | **0.39 [0.25, 0.61]** (6) |
|  | **By Route** | Oral  Oral and/or Enteral/Parenteral  Enteral | NA  NA  NA | 0.27 [0.03, 2.64] (2)  NA  **0.40 [0.26, 0.63]** (3) |
|  | **By Time** | Preoperative  Preoperative and Intra/Postoperative  Intra/Postoperative | NA  NA  NA | NA  0.24 [0.02, 2.27] (2)  **0.40 [0.26, 0.63]** (3) |
|  | **By Country** | Europe  Asia  North America  South America | NA  NA  NA  NA | 0.27 [0.03, 2.64] (2)  **0.40 [0.25, 0.62]** (4)  NA  NA |
|  | **By Age** | ≤ 65 years  > 65 years | NA  NA | 0.46 [0.11, 1.96] (4)  0.34 [0.16, 0.73] (2) |
|  | **By Product** | Impact  Not Reported | NA  NA | 0.46 [0.11, 1.96] (4)  **0.34 [0.16, 0.73]** (2) |

NA = not available

**Table 4. Impact of Probiotics supplementation on postoperative infectious complications.**

| **Outcome** |  | **Subgroup analysis** | **RCT** |
| --- | --- | --- | --- |
|  |  |  | **OR [95% CI]; (N. Studies)** |
| **Infectious complication** | **All** |  | 0.53 [0.13, 2.09] (2) |
| **Surgical Site Infection (SSI)** | **All** |  | **0.61 [0.42, 0.91]** (7) |
|  | **By Route** | Oral  Oral and/or Enteral/Parenteral  Enteral | 0.79 [0.48, 1.32] (5)  NA  NA |
|  | **By Time** | Preoperative  Preoperative and Intra/Postoperative  Intra/Postoperative | **0.47 [0.22, 0.99]** (2)  0.68 [0.43, 1.07] (5)  NA |
|  | **By Country** | Europe  Asia  North America  South America | **0.42 [0.22, 0.78]** (2)  0.79 [0.48, 1.32] (5)  NA  NA |
|  | **By Age** | ≤ 65 years  > 65 years | 0.61 [0.32, 1.15] (3)  0.60 [0.27, 1.33] (3) |
|  | **By Product** | LactoLevure  Not Reported | **0.42 [0.22, 0.78]** (2)  0.79 [0.48, 1.32] (5) |
| **Bloodstream Infection (BSI)** | **All** |  | **0.42 [0.18, 0.98]** (5) |
|  | **By Route** | Oral  Oral and/or Enteral/Parenteral  Enteral | 0.34 [0.11, 1.02] (4)  NA  NA |
|  | **By Time** | Preoperative  Preoperative and Intra/Postoperative  Intra/Postoperative | NA  0.52 [0.22, 1.24] (4)  NA |
|  | **By Country** | Europe  Asia  North America  South America | NA  0.34 [0.11, 1.02] (4)  NA  NA |
|  | **By Product** | LactoLevure  Not Reported | NA  0.34 [0.11, 1.02] (4) |
|  | **By Disease** | Cancer  Other | **0.42 [0.18, 0.98]** (5)  NA |
| **Urinary Tract Infections (UTI** | **All** |  | **0.32 [0.14, 0.74]** (4) |
|  | **By Route** | Oral  Oral and/or Enteral/Parenteral  Enteral | 0.25 [0.06, 1.05] (3)  NA  NA |
|  | **By Time** | Preoperative  Preoperative and Intra/Postoperative  Intra/Postoperative | **0.32 [0.14, 0.74]** (4)  NA  NA |
|  | **By Country** | Europe  Asia  North America  South America | NA  0.25 [0.06, 1.05] (3)  NA  NA |
|  | **By Age** | ≤ 65 years  > 65 years | 0.32 [0.03, 3.05] (2)  0.38 [0.13, 1.12] (2) |
|  | **By Produc** | LactoLevure  Not Reported | NA  0.25 [0.06, 1.05] (3) |
| **Pneumonia** | **All** |  | **0.42 [0.22, 0.81]** (5) |
|  | **By Route** | Oral  Oral and/or Enteral/Parenteral  Enteral | 0.52 [0.25, 1.08] (4)  NA  NA |
|  | **By Time** | Preoperative  Preoperative and Intra/Postoperative  Intra/Postoperative | NA  **0.45 [0.22, 0.89]** (4)  NA |
|  | **By Country** | Europe  Asia | NA  0.52 [0.25, 1.08] (4) |
|  | **By Age** | ≤ 65 years  > 65 years | 0.67 [0.27, 1.66] (2)  **0.26 [0.08, 0.80]** (2) |
|  | **By Product** | LactoLevure  Not Reported | NA  0.52 [0.25, 1.08] (4) |

NA = not available

**Table 5. Impact of Synbiotics supplementation on postoperative infectious complications.**

| **Outcome** |  | **Subgroup analysis** | **RCT** |
| --- | --- | --- | --- |
|  |  |  | **OR [95% CI] (N. Studies)** |
| **Infectious complication** | **All** |  | 0.41 [0.15, 1.11] (5) |
|  | **By Route** | Oral  Oral and/or Enteral/Parenteral  Enteral | **0.33 [0.12, 0.96]** (3)  0.58 [0.04, 7.70] (2)  NA |
|  | **By Time** | Preoperative  Preoperative and Intra/Postoperative  Intra/Postoperative | NA  0.42 [0.12, 1.52] (4)  NA |
|  | **By Country** | Europe  Asia  North America  South America | NA  0.71 [0.25, 2.00] (3)  NA  NA |
|  | **By Age** | ≤ 65 years  > 65 years | **0.18 [0.08, 0.42]** (3)  NA |
|  | **By Product** | Yakult  Not Reported | 0.71 [0.25, 2.00] (3)  **0.19 [0.07, 0.56]** (2) |
| **Surgical Site Infection (SSI)** | **All** |  | 0.54 [0.26, 1.11] (5) |
|  | **By Route** | Oral  Oral and/or Enteral/Parenteral  Enteral | 0.34 [0.12, 1.00] (3)  1.76 [0.39, 7.91] (2)  NA |
|  | **By Time** | Preoperative  Preoperative and Intra/Postoperative  Intra/Postoperative | NA  0.54 [0.26, 1.11] (5)  NA |
|  | **By Country** | Europe  Asia | NA  0.65 [0.37, 1.14] (4) |
|  | **By Age** | ≤ 65 years  > 65 years | **0.17 [0.06, 0.54]** (2)  NA |
|  | **By Product** | Yakult  Not Reported | 0.77 [0.42, 1.43] (3)  **0.17 [0.06, 0.54]** (2) |
| **Bloodstream Infection (BSI)** | **All** |  | 0.95 [0.54, 1.66] (5) |
|  | **By Route** | Oral  Oral and/or Enteral/Parenteral  Enteral | 0.89 [0.48, 1.66] (3)  1.27 [0.21, 7.80] (2)  NA |
|  | **By Time** | Preoperative  Preoperative and Intra/Postoperative  Intra/Postoperative | NA  1.04 [0.56, 1.92] (4)  NA |
|  | **By Country** | Europe  Asia | 0.94 [0.50, 1.77] (2)  0.99 [0.30, 3.25] (3) |
|  | **By Age** | ≤ 65 years  > 65 years | 0.55 [0.16, 1.90] (2)  NA |
|  | **By Product** | Yakult  Not Reported | 1.27 [0.21, 7.80] (2)  0.89 [0.48, 1.66] (3) |
| **Pneumonia** | **All** |  | 0.71 [0.33, 1.55] (5) |
|  | **By Route** | Oral  Oral and/or Enteral/Parenteral  Enteral | 0.42 [0.15, 1.23] (3)  1.47 [0.43, 4.96] (2)  NA |
|  | **By Time** | Preoperative  Preoperative and Intra/Postoperative  Intra/Postoperative | NA  0.72 [0.30, 1.73] (4)  NA |
|  | **By Country** | Europe  Asia | NA  1.14 [0.42, 3.11] (3) |
|  | **By Age** | ≤ 65 years  > 65 years | 0.42 [0.15, 1.23] (3)  NA |
|  | **By Product** | Yakult  Not Reported | 1.47 [0.43, 4.96] (2)  0.42 [0.15, 1.23] (3) |

NA = not available

**Table 6. Impact of Protein Integration on postoperative infectious complications.**

| **Outcome** | **Subgroup analysis** | | **RCT** |
| --- | --- | --- | --- |
|  |  |  | **OR [95% CI]; (N. Studies)** |
| **Surgical Site Infection (SSI)** | **All** |  | **0.36 [0.17, 0.75]** (4) |
|  | **By Route** | Oral  Oral and/or Enteral/Parenteral  Parenteral | **0.31 [0.14, 0.69]** (2)  NA  0.95 [0.12, 7.23] (2) |
|  | **By Time** | Preoperative  Preoperative and Intra/Postoperative  Intra/Postoperative | NA  NA  0.28 [0.07, 1.06] (3) |
|  | **By Country** | Europe  Asia  North America  South America | NA  0.95 [0.12, 7.23] (2)  NA  NA |
|  | **By Age** | ≤ 65 years  ≥ 65 years | 0.28 [0.07, 1.06] (3)  NA |
|  | **By Product** | Yakult  Not Reported | NA  **0.36 [0.17, 0.75]** (4) |
| **Bloodstream Infection (BSI)** | **All** |  | 0.26 [0.01, 4.64] (2) |
| **Urinary Tract Infection (UTI)** | **All** |  | 0.52 [0.22, 1.27] (3) |
| **Pneumonia** | **All** |  | 0.91 [0.18, 4.64] (3) |

NA = not available

**Table 7. Effect of Nutritional Supplementation on Hospital Length of Stay.**

| **Supplementation** | **Subgroup analysis** | **RCT** | **Analytical** |
| --- | --- | --- | --- |
|  |  | **Mean Diff. [95% CI]; (N. Studies)** | **Mean Diff. [95% CI]; (N. Studies)** |
| **Immunonutrition** | **All**  Postoperative LOS | **-2.64 [-5.24, -0.03]** (5)  -2.70 [-6.85, 1.44] (4) | 1.21 [-6.13, 8.55] (5)  NA |
| **Probiotic** | **All**  Postoperative LOS | -0.94 [-2.07, 0.20] (4)  -0.58 [-1.97, 0.81] (3) | NA |
| **Synbiotic** | **All**  Postoperative LOS | **-9.25 [-11.02, -7.47]** (6)  -4.05 [-14.01, 5.91] (4) | NA |
| **Protein Integration** | **All** | -4.64 [-15.88, 6.61] (2) | NA |

NA = not available

**Table 8. Nutritional intervention compared to standard of care for preventing UTI**

| **Outcomes** | **Anticipated absolute effects* (95% CI)** | | **Relative effect (95% CI)** | **№ of participants (studies)** | **Certainty of the evidence (GRADE)** | **Comments** |
| --- | --- | --- | --- | --- | --- | --- |
|  | **Risk with standard of care** | **Risk with intervention** |  |  |  |  |
| **Probiotics** | 101 per 1.000 | 35 per 1.000 (15 to 77) | OR 0.32 (0.14 to 0.74) | 458 (4 RCTs) | ⨁⨁◯◯ Low^a^ | Probiotics may result in a large reduction in UTI. |
| **Protein integration** | 203 per 1.000 | 117 per 1.000 (53 to 244) | OR 0.52 (0.22 to 1.27) | 159 (3 RCTs) | ⨁◯◯◯ Very low^b,c,d^ | The evidence is very uncertain about the effect of protein Integration on UTI. |

*The risk in the intervention group (and its 95% confidence interval) is based on the assumed risk in the comparison group and the relative effect of the intervention (and its 95% CI). CI: confidence interval; MD: mean difference; OR: odds ratio; a. Downgraded by one level for wide confidence intervals that include both "important" and "no effect"; b. Downgraded by one level for high risk of bias in different studies in multiple critical domains; c. Downgraded by one level for difference in kind of surgery; d. Downgraded by one level for not optimal information size and confidence intervals included both "important" and "no effect";

**Table 9. Nutritional intervention compared to standard of care for preventing Pneumonia**

| **Outcomes** | **Anticipated absolute effects* (95% CI)** | | **Relative effect (95% CI)** | **№ of participants (studies)** | **Certainty of the evidence (GRADE)** | **Comments** |
| --- | --- | --- | --- | --- | --- | --- |
|  | **Risk with standard of care** | **Risk with intervention** |  |  |  |  |
| **Probiotics** | 120 per 1.000 | 54 per 1.000 (29 to 100) | OR 0.42 (0.22 to 0.81) | 518 (5 RCTs) | ⨁⨁◯◯ Low^a^ | Probiotics may result in a large reduction in pneumonia. |
| **Symbiotic** | 118 per 1.000 | 87 per 1.000 (42 to 172) | OR 0.71 (0.33 to 1.55) | 295 (5 RCTs) | ⨁⨁⨁◯ Moderate^b^ | Synbiotics likely results in little to no difference in pneumonia. |
| **Protein integration** | 216 per 1.000 | 201 per 1.000 (47 to 561) | OR 0.91 (0.18 to 4.64) | 159 (3 RCTs) | ⨁◯◯◯ Very low^b,d,e,f^ | The evidence is very uncertain about the effect of protein Integration on pneumonia. |

*The risk in the intervention group (and its 95% confidence interval) is based on the assumed risk in the comparison group and the relative effect of the intervention (and its 95% CI). CI: confidence interval; MD: mean difference; OR: odds ratio; a. Downgraded by one level for wide confidence intervals that include both "important" and "no effect"; b. Downgraded by one level for not optimal information size and confidence intervals included both "important" and "no effect"; d. Downgraded by one level for high risk of bias in different studies in multiple critical domains; e. Downgraded by one level for difference in kind of surgery; f. Downgraded by one level for serious inconsistency. Evidence of moderate heterogeneity (I2=69.43%), with only partial overlap of confidence intervals and studies showing different effects of intervention

**Table 10. Nutritional intervention compared to standard of care for preventing Hospital Length of Stay (LOS)**

| **Outcomes** | **Anticipated absolute effects***  **(95% CI)** | | **№ of participants (studies)** | **Certainty of the evidence (GRADE)** | **Comments** |
| --- | --- | --- | --- | --- | --- |
|  | **Risk with standard of care** | **Risk with intervention** |  |  |  |
| **Immunonutrition** | Mean hospital LOS 18.12 days | MD 2.64 days fewer (5.24 fewer to  0.03 fewer) | 682 (5 RCTs) | ⨁⨁◯◯ Low^a,b^ | Immunonutrition may reduce hospital Lenght of Stay slightly. |
| **Probiotics** | Mean hospital LOS 13.7 days | MD 0.94 days fewer (2.07 fewer to 0.2 more) | 354 (4 RCTs) | ⨁⨁◯◯ Low^c,d^ | Probiotics may result in little to no difference in hospital Lenght of Stay. |
| **Synbiotics** | Mean hospital LOS 32.4 days | MD 9.25 days fewer (11.02 fewer to 7.47 fewer) | 297 (6 RCTs) | ⨁⨁⨁◯ Moderate^e^ | Synbiotics probably results in a large reduction in hospital Lenght of Stay. |

*The risk in the intervention group (and its 95% confidence interval) is based on the assumed risk in the comparison group and the relative effect of the intervention (and its 95% CI). CI: confidence interval; MD: mean difference; OR: odds ratio; a. Downgraded by one level for not optimal information size; b. Downgraded by one level for high heterogeneity: I2 was 85.74%; c. Downgraded by one level for high heterogeneity and IC95% and only partially overlapping; d. Downgraded by one level for wide confidence intervals that include both "important" and "no effect"; e. Downgraded by one level for not optimal information size, only one big study with a weight of 88.11% while the others contributed for a very small part of the analysis

# Supplementary Figures

**Figure 1.** Traffic Light Plot of Risk of Bias for RCTs (A), observational cohort studies (B) and case control studies (C).


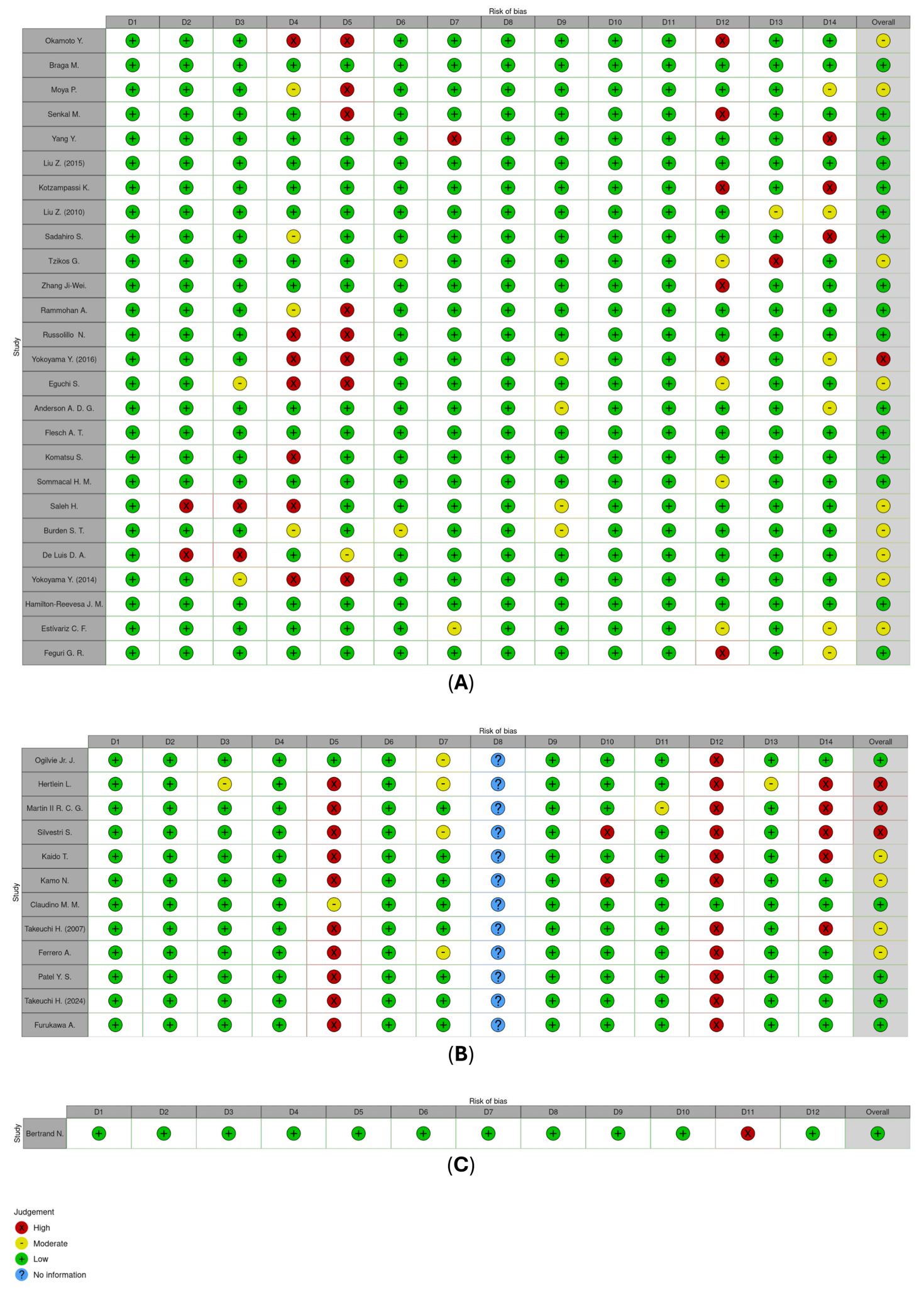


(**A**) D1: Was the study described as randomized, a randomized trial, a randomized clinical trial, or an RCT? D2: Was the method of randomization adequate (i.e., use of randomly generated assignment)? D3: Was the treatment allocation concealed (so that assignments could not be predicted)? D4: Were study participants and providers blinded to treatment group assignment? D5: Were the people assessing the outcomes blinded to the participants' group assignments? D6: Were the groups similar at baseline on important characteristics that could affect outcomes (e.g., demographics, risk factors, co-morbid conditions)? D7. Was the overall drop-out rate from the study at endpoint 20% or lower of the number allocated to treatment? D8: Was the differential drop-out rate (between treatment groups) at endpoint 15 percentage points or lower? D9: Was there high adherence to the intervention protocols for each treatment group? D10: Were other interventions avoided or similar in the groups (e.g., similar background treatments)? D11: Were outcomes assessed using valid and reliable measures, implemented consistently across all study participants? D12: Did the authors report that the sample size was sufficiently large to be able to detect a difference in the main outcome between groups with at least 80% power? D13: Were outcomes reported or subgroups analyzed prespecified (i.e., identified before analyses were conducted)? D14: Were all randomized participants analyzed in the group to which they were originally assigned, i.e., did they use an intention-to-treat analysis?

(**B**) D1: Was the research question or objective in this paper clearly stated? D2: Was the study population clearly specified and defined? D3: Was the participation rate of eligible persons at least 50%? D4: Were all the subjects selected or recruited from the same or similar populations (including the same time period)? Were inclusion and exclusion criteria for being in the study prespecified and applied uniformly to all participants? D5: Was a sample size justification, power description, or variance and effect estimates provided? D6: For the analyses in this paper, were the exposure(s) of interest measured prior to the outcome(s) being measured? D7: Was the timeframe sufficient so that one could reasonably expect to see an association between exposure and outcome if it existed? D8: For exposures that can vary in amount or level, did the study examine different levels of the exposure as related to the outcome (e.g., categories of exposure, or exposure measured as continuous variable)? D9: Were the exposure measures (independent variables) clearly defined, valid, reliable, and implemented consistently across all study participants? D10: Was the exposure(s) assessed more than once over time? D11: Were the outcome measures (dependent variables) clearly defined, valid, reliable, and implemented consistently across all study participants? D12: Were the outcome assessors blinded to the exposure status of participants? D13: Was loss to follow-up after baseline 20% or less? D14: Were key potential confounding variables measured and adjusted statistically for their impact on the relationship between exposure(s) and outcome(s)?

(**C**) D1: Was the research question or objective in this paper clearly stated and appropriate? D2: Was the study population clearly specified and defined? D3: Did the authors include a sample size justification? D4: Were controls selected or recruited from the same or similar population that gave rise to the cases (including the same timeframe)? D5: Were the definitions, inclusion and exclusion criteria, algorithms or processes used to identify or select cases and controls valid, reliable, and implemented consistently across all study participants? D6: Were the cases clearly defined and differentiated from controls? D7: If less than 100 percent of eligible cases and/or controls were selected for the study, were the cases and/or controls randomly selected from those eligible? D8: Was there use of concurrent controls? D9: Were the investigators able to confirm that the exposure/risk occurred prior to the development of the condition or event that defined a participant as a case? D10: Were the measures of exposure/risk clearly defined, valid, reliable, and implemented consistently (including the same time period) across all study participants? D11: Were the assessors of exposure/risk blinded to the case or control status of participants? D12: Were key potential confounding variables measured and adjusted statistically in the analyses? If matching was used, did the investigators account for matching during study analysis?

**Figure 2.** Summary Plot of Risk of Bias for RCTs (A), observational cohort studies (B) and case control studies (C).


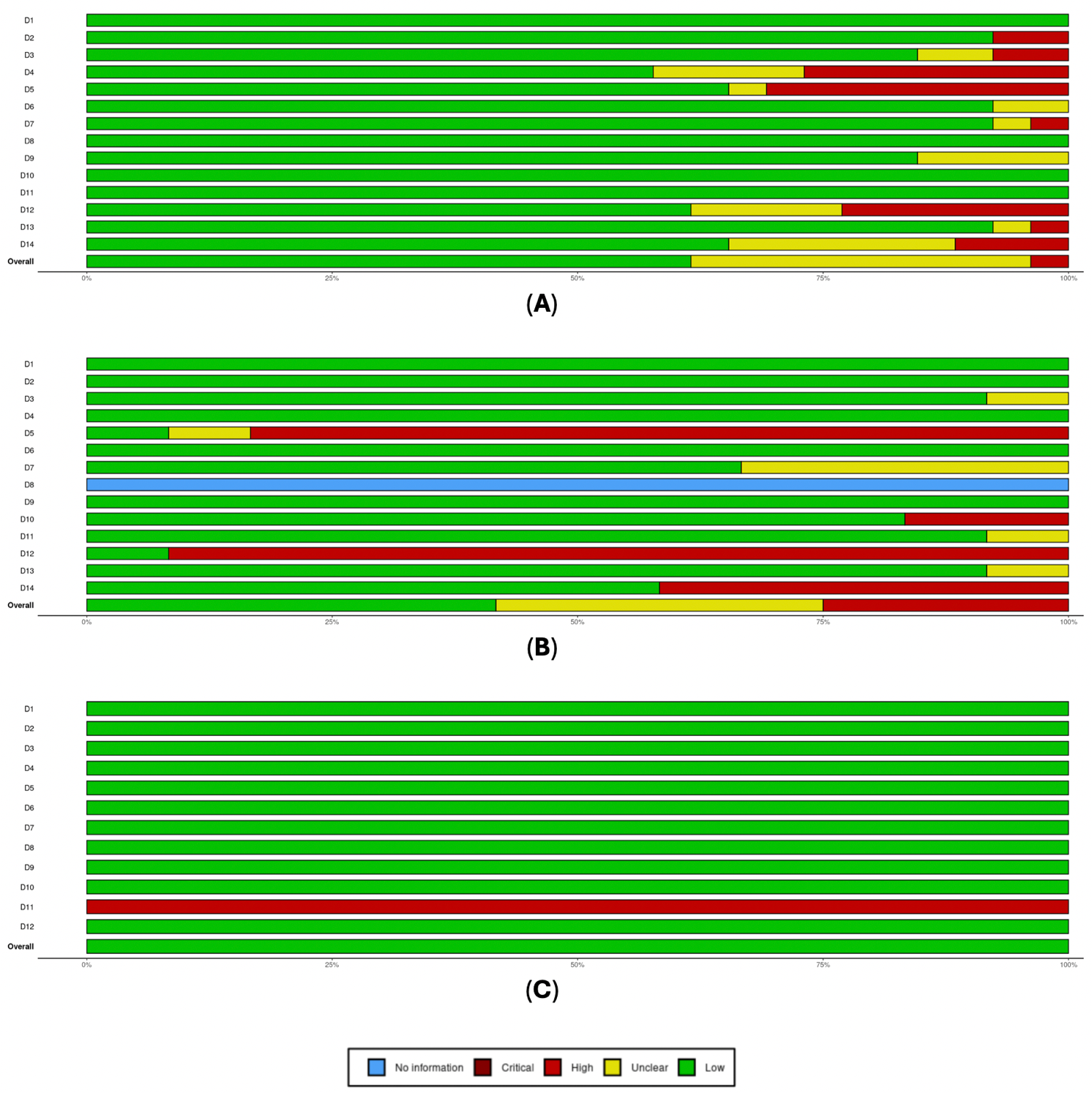


**Figure 3.** Forest plot showing the effect of Probiotic on Bloodstream Infections for RCT studies.


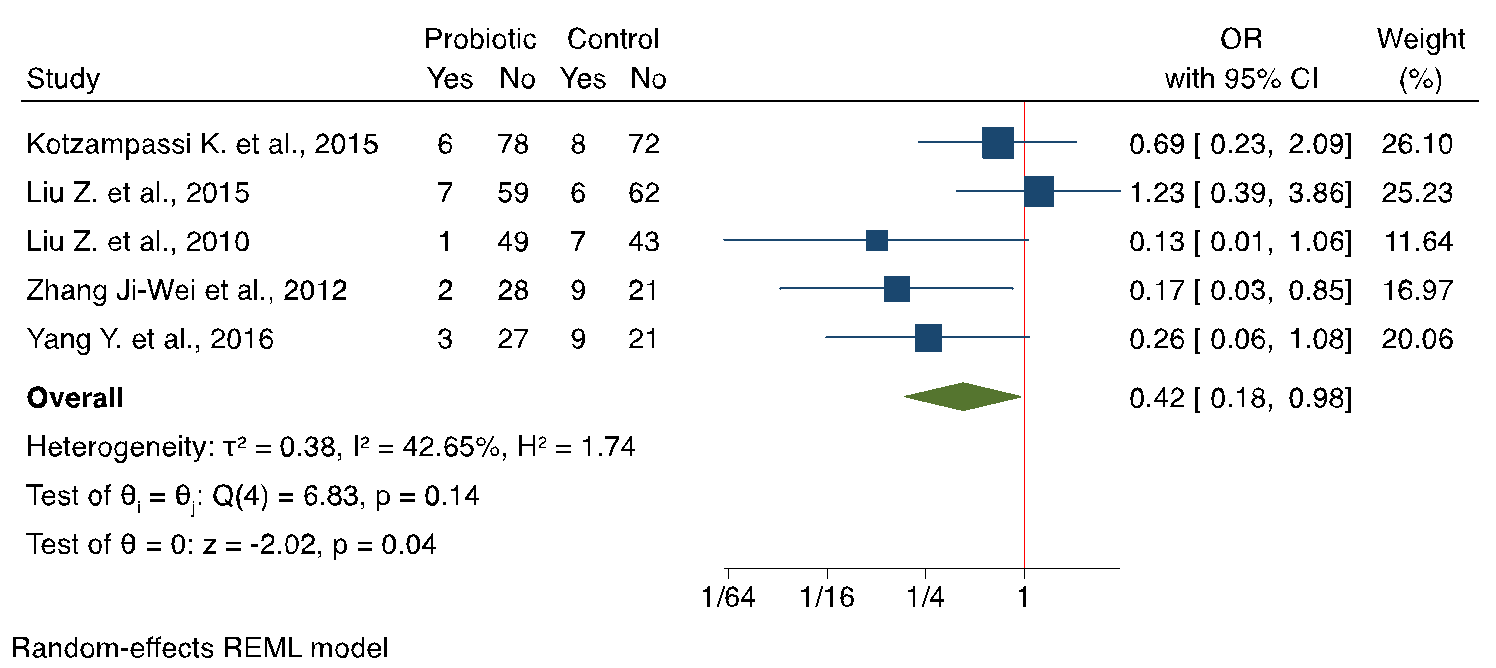


**Figure 4.** Forest plot showing the effect of Immunonutrition on Bloodstream Infections for Analytical studies.


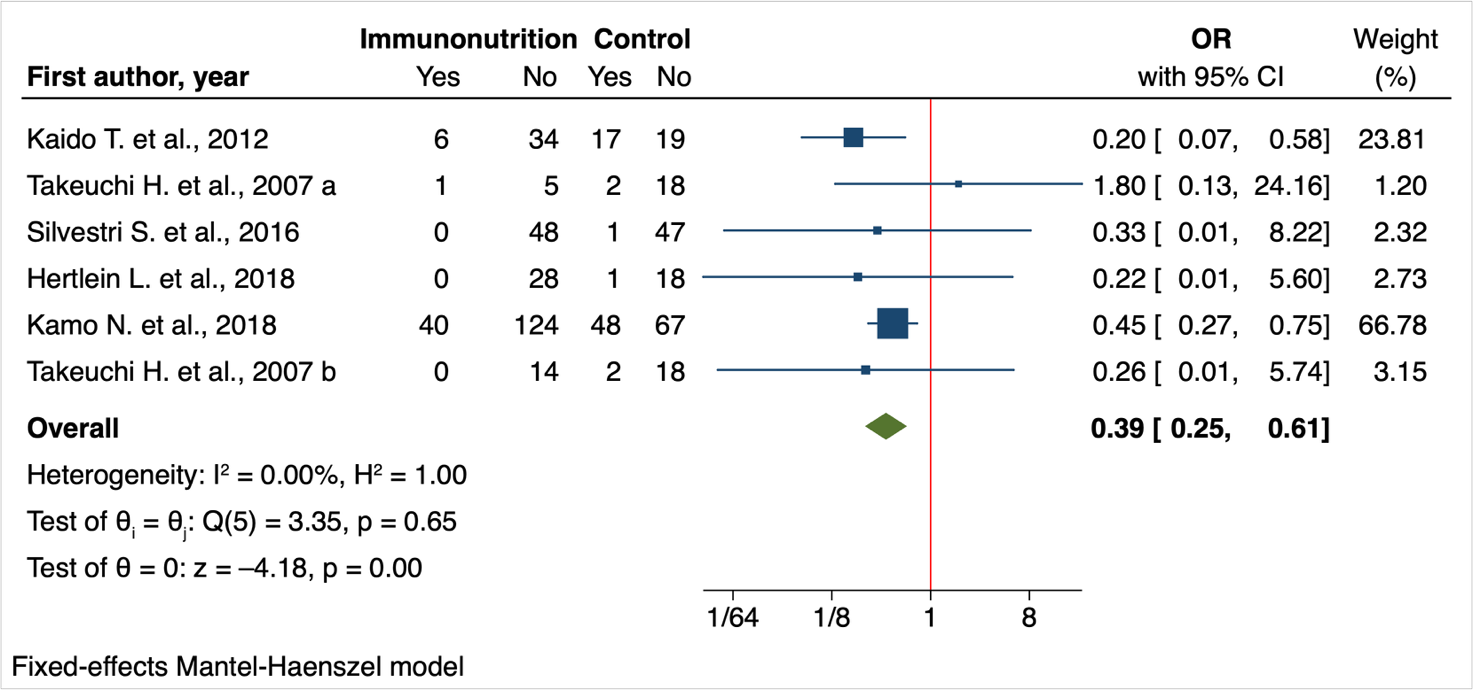


**Figure 5.** Forest plot showing the effect of Probiotic on Urinary Tract Infections for RCT studies.


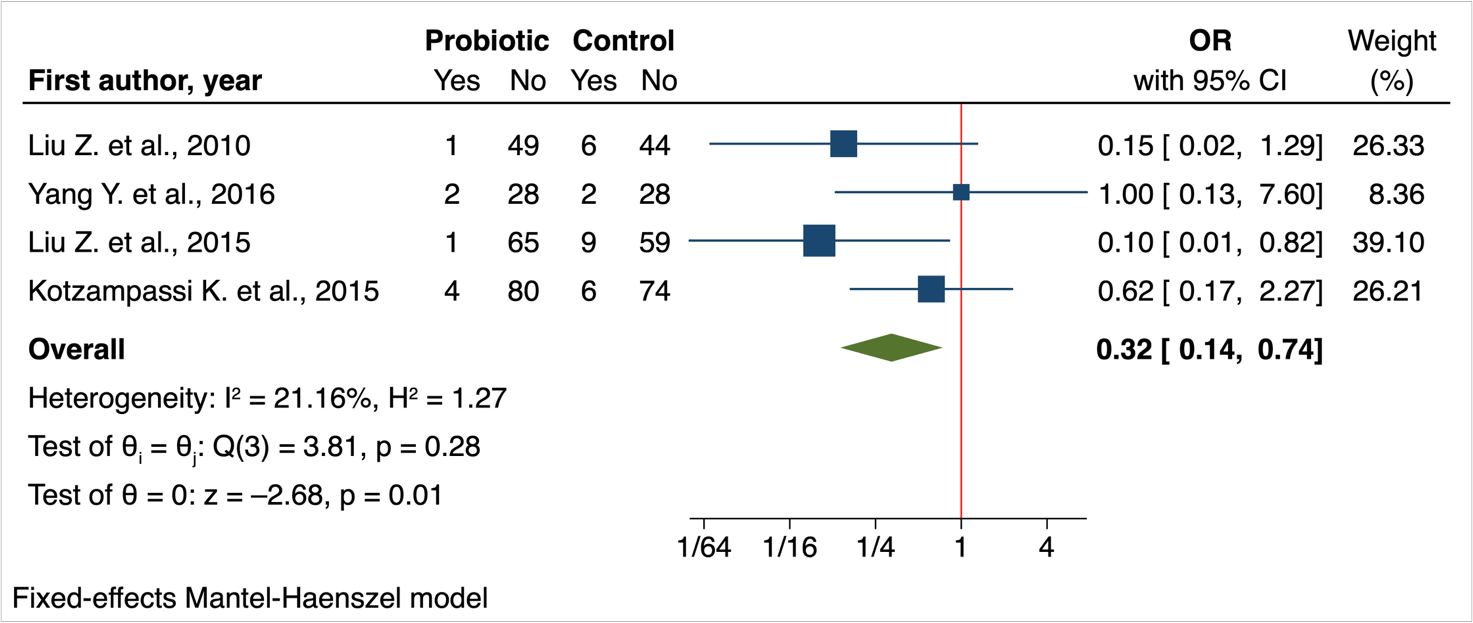


**Figure 6.** Forest plot showing the effect of Probiotic on Pneumonias for RCT studies.


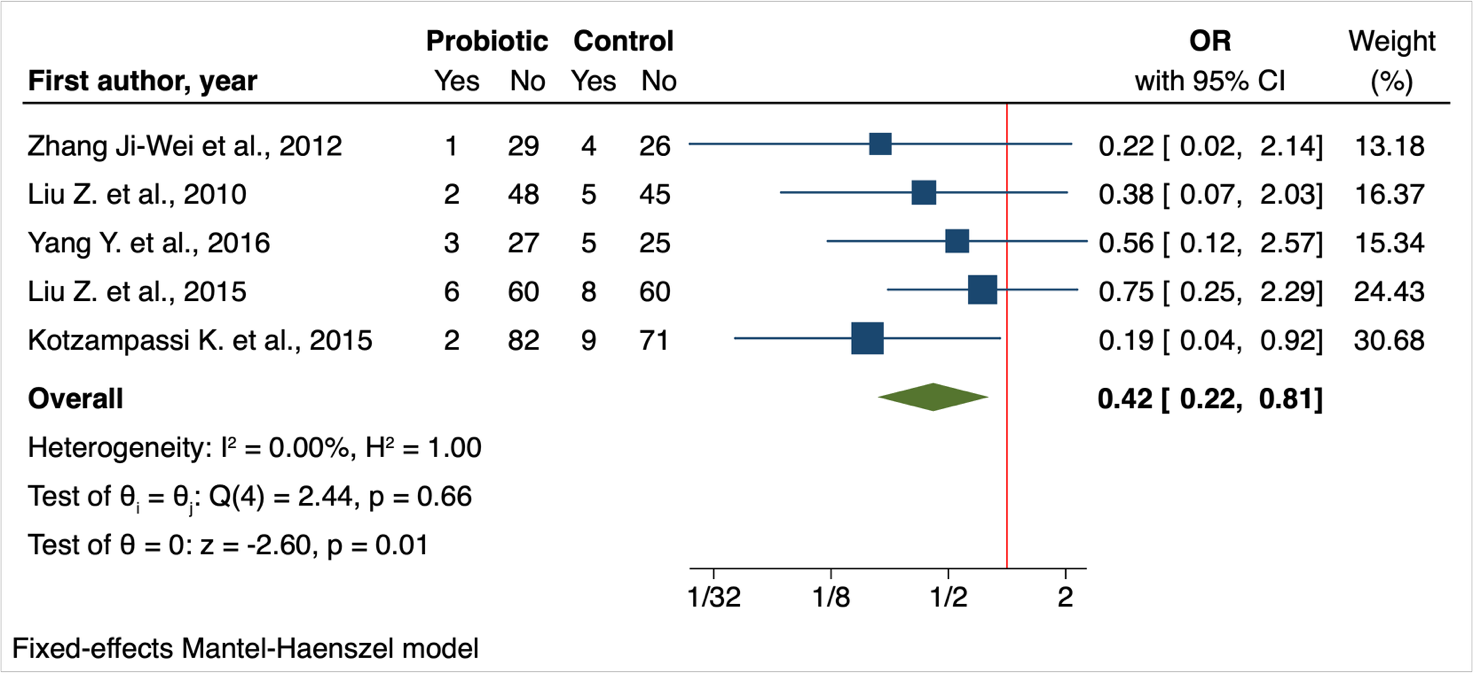


**Figure 7.** Forest plot showing the effect of Immunonutrition on Hospital Length of Stay for RCT studies.


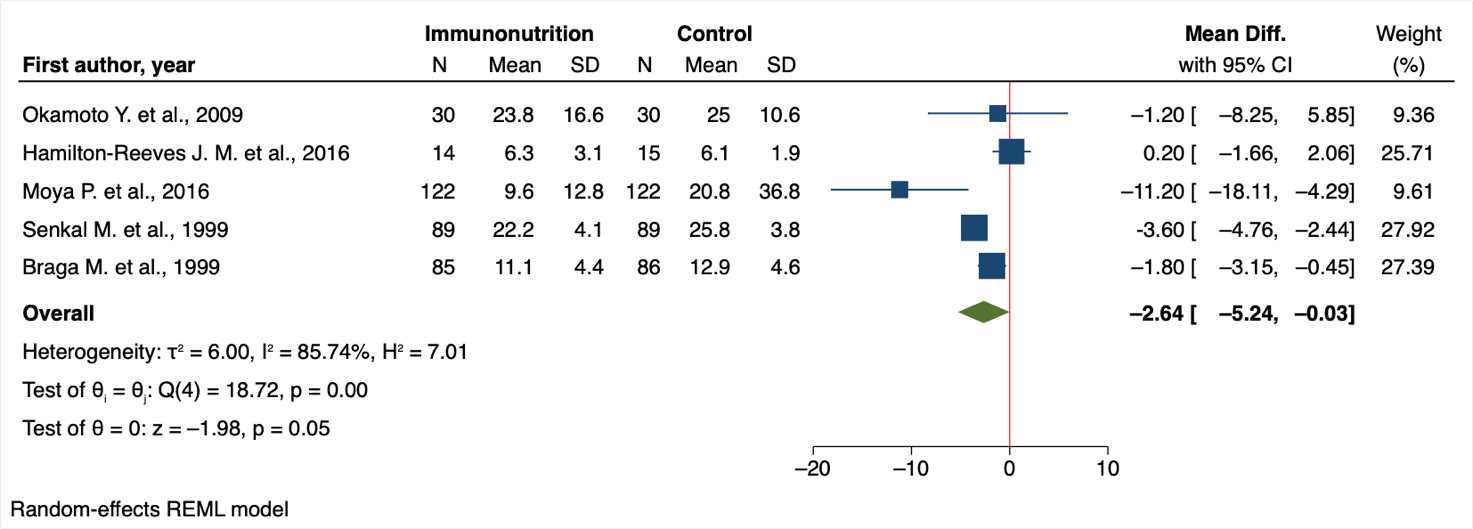


**Figure 8.** Forest plot showing the effect of synbiotics on Hospital Length of Stay for RCT studies.


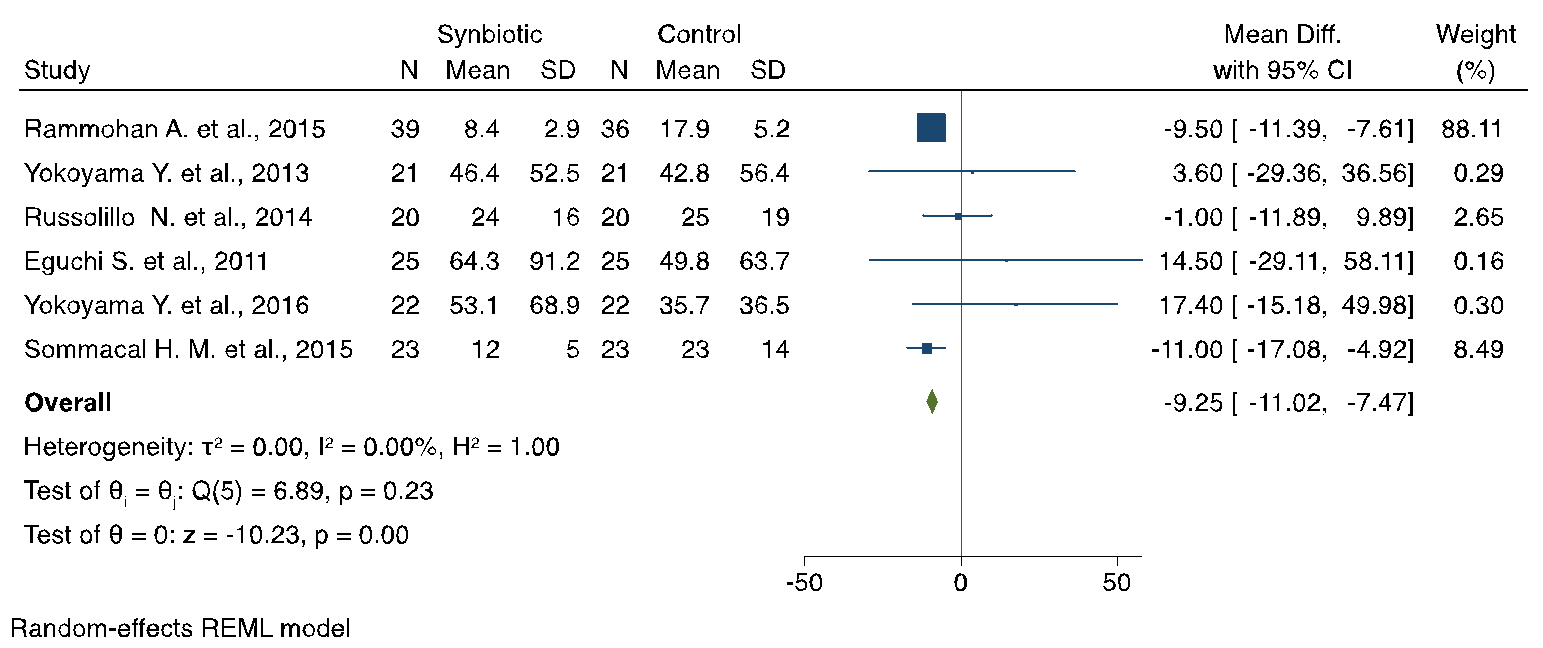

Supplement: Supplementary file 1 [file Data_Sheet_1.ZIP › Supplementary_Material SARTINI M et al.docx]
